# Assembly and annotation of the black spruce genome provide insights on spruce phylogeny and evolution of stress response

**DOI:** 10.1093/g3journal/jkad247

**Published:** 2023-10-24

**Authors:** Theodora Lo, Lauren Coombe, Kristina K Gagalova, Alex Marr, René L Warren, Heather Kirk, Pawan Pandoh, Yongjun Zhao, Richard A Moore, Andrew J Mungall, Carol Ritland, Nathalie Pavy, Steven J M Jones, Joerg Bohlmann, Jean Bousquet, Inanç Birol, Ashley Thomson

**Affiliations:** Canada’s Michael Smith Genome Sciences Centre, BC Cancer, Vancouver, BC V5Z 4S6, Canada; Canada’s Michael Smith Genome Sciences Centre, BC Cancer, Vancouver, BC V5Z 4S6, Canada; Canada’s Michael Smith Genome Sciences Centre, BC Cancer, Vancouver, BC V5Z 4S6, Canada; Canada’s Michael Smith Genome Sciences Centre, BC Cancer, Vancouver, BC V5Z 4S6, Canada; Canada’s Michael Smith Genome Sciences Centre, BC Cancer, Vancouver, BC V5Z 4S6, Canada; Canada’s Michael Smith Genome Sciences Centre, BC Cancer, Vancouver, BC V5Z 4S6, Canada; Canada’s Michael Smith Genome Sciences Centre, BC Cancer, Vancouver, BC V5Z 4S6, Canada; Canada’s Michael Smith Genome Sciences Centre, BC Cancer, Vancouver, BC V5Z 4S6, Canada; Canada’s Michael Smith Genome Sciences Centre, BC Cancer, Vancouver, BC V5Z 4S6, Canada; Canada’s Michael Smith Genome Sciences Centre, BC Cancer, Vancouver, BC V5Z 4S6, Canada; Department of Forest and Conservation Sciences, University of British Columbia, Vancouver, BC V6T 1Z4, Canada; Michael Smith Laboratories, University of British Columbia, Vancouver, BC V6T 1Z4, Canada; Canada Research Chair in Forest Genomics, Laval University, Quebec City, QC G1V 0A6, Canada; Canada’s Michael Smith Genome Sciences Centre, BC Cancer, Vancouver, BC V5Z 4S6, Canada; Department of Forest and Conservation Sciences, University of British Columbia, Vancouver, BC V6T 1Z4, Canada; Michael Smith Laboratories, University of British Columbia, Vancouver, BC V6T 1Z4, Canada; Department of Botany, University of British Columbia, Vancouver, BC V6T 1Z4, Canada; Canada Research Chair in Forest Genomics, Laval University, Quebec City, QC G1V 0A6, Canada; Canada’s Michael Smith Genome Sciences Centre, BC Cancer, Vancouver, BC V5Z 4S6, Canada; Faculty of Natural Resources Management, Lakehead University, Thunder Bay, ON P7B 5E1, Canada

**Keywords:** genome assembly and annotation, black spruce, *Picea mariana*, conifer, gymosperm

## Abstract

Black spruce (*Picea mariana* [Mill.] B.S.P.) is a dominant conifer species in the North American boreal forest that plays important ecological and economic roles. Here, we present the first genome assembly of *P. mariana* with a reconstructed genome size of 18.3 Gbp and NG50 scaffold length of 36.0 kbp. A total of 66,332 protein-coding sequences were predicted in silico and annotated based on sequence homology. We analyzed the evolutionary relationships between *P. mariana* and 5 other spruces for which complete nuclear and organelle genome sequences were available. The phylogenetic tree estimated from mitochondrial genome sequences agrees with biogeography; specifically, *P. mariana* was strongly supported as a sister lineage to *P. glauca* and 3 other taxa found in western North America, followed by the European *Picea abies*. We obtained mixed topologies with weaker statistical support in phylogenetic trees estimated from nuclear and chloroplast genome sequences, indicative of ancient reticulate evolution affecting these 2 genomes. Clustering of protein-coding sequences from the 6 *Picea* taxa and 2 *Pinus* species resulted in 34,776 orthogroups, 560 of which appeared to be specific to *P. mariana*. Analysis of these specific orthogroups and d*N*/d*S* analysis of positive selection signatures for 497 single-copy orthogroups identified gene functions mostly related to plant development and stress response. The *P. mariana* genome assembly and annotation provides a valuable resource for forest genetics research and applications in this broadly distributed species, especially in relation to climate adaptation.

## Introduction

Globally and particularly within the boreal biome, forest health is declining due to the vulnerability of trees to increasing biotic and abiotic stresses associated with climate change (Allen *et al.* 2010, 2015; Gauthier *et al.* 2015). While some tree species or populations may have the capacity for rapid adaptation or migration, others will suffer from maladaptation under changing environmental conditions (Aitken *et al.* 2008; Benomar *et al.* 2022). One species of interest is black spruce (*Picea mariana* [B.S.P.] Mill.), as it is both an ecologically and economically important conifer, being one of the most abundant, widely planted tree species in Canada's boreal forests and highly valued for its wood products (Mullin *et al.* 2011). As a transcontinental species, geographic genetic variation related to phylogeographic history (Jaramillo-Correa *et al.* 2004; Gérardi *et al.* 2010; Prunier *et al.* 2012) and adaptive variation of clinal nature in relation to climate have been reported (Beaulieu *et al.* 2004; Thomson *et al.* 2009). Given the pace of climate change at boreal latitudes, significant maladaptation is expected to occur across much of the species’ range (Thomson *et al.* 2009).

Early studies have played a critical role in advancing our understanding of the genomic basis of adaptive variation in forest trees (Neale and Kremer 2011; Plomion *et al.* 2016). The sequencing of the first conifer giga-genomes, including *Picea glauca* (Birol *et al.* 2013; Warren, Keeling, *et al.* 2015), *Picea abies* (Nystedt *et al.* 2013), *Pinus taeda* (Neale *et al.* 2014), *Pinus lambertiana* (Gonzalez-Ibeas *et al.* 2016), *Picea engelmannii* (Gagalova *et al.* 2022), *Picea sitchensis* (Gagalova *et al.* 2022), and more recently, the chromosome-scale assemblies of *Sequoiadendron giganteum* (Scott *et al.* 2020), *Pinus tabuliformis* (Niu *et al.* 2022), and *Sequoia sempervirens* (Neale *et al.* 2022), provided key insights into the different genome structure and evolution of these gymnosperm species. Other significant genomic resources have been developed for spruces, although they are limited to transcriptome-related data for various species (Bousquet *et al.* 2021). For black spruce, saturated genetic maps, annotated gene, and SNP resources, and a gene-based genotyping chip have been made available (Pavy *et al.* 2008; Mann *et al.* 2013; Pavy *et al.* 2016; Van Ghelder *et al.* 2019), enabling association genetics and quantitative trait loci studies (Prunier *et al.* 2011, 2013), as well as the deployment of genomic selection (Lenz *et al.* 2017). The chloroplast genome sequence of black spruce has also been more recently determined (Lo *et al.* 2020).

Complete conifer genome sequences have been sparse due to the associated high cost of sequencing and atypical genome sizes which are amongst the largest of all plants—typically around 20 Gb (De La Torre *et al.* 2014). However, the rapidly decreasing cost of high-throughput sequencing has recently enabled genome sequencing and assembly for many tree species, with more than 30 genome assemblies now available, including the large genomes of 4 economically important North American spruces (Falk *et al.* 2018; Gagalova *et al.* 2022). The burgeoning availability of genomic resources has facilitated an increase in the number of genome-wide population genetic studies of conifers, providing knowledge on the genomic architecture of adaptive variation necessary for improved breeding and conservation under climate change (Namroud *et al.* 2008; Pelgas *et al.* 2011; Chen *et al.* 2012; Savolainen *et al.* 2013; Hornoy *et al.* 2015; Yeaman *et al.* 2016; Depardieu *et al.* 2021; MacLachlan *et al.* 2021). These genomic resources have also hastened the development and deployment of tree breeding strategies directly tackling stress response in relation to climate change (Isabel *et al.* 2020; Laverdière *et al.* 2022).

Given that much of the black spruce genome has remained undeciphered, its sequencing, assembly, and annotation will be a valuable contribution to the community. The new black spruce genomic resources will facilitate future studies into natural genetic variation and trait genomic architecture in relation to local adaptation, thereby enabling improved conservation and breeding strategies under climate change. Here, we present the first reference genome for black spruce, its phylogenetic implications, and analyze selection signatures in relation to adaptation to biotic and abiotic stress.

## Materials and methods

### DNA extraction and sequencing

An individual tree that provided convenient access to newly flushed bud tissue was selected from a long-term black spruce provenance test in Thunder Bay, Ontario. The selected individual (genotype 40-10-1) represents a provenance native to northwestern Ontario (50° 57′ 39.96′′N, 90° 27′ 20.16′′E; elevation, 741 m). Sampled tissue was immediately flash-frozen in liquid nitrogen and maintained at −80°C until the time of DNA extraction. High molecular weight (HMW) DNA was extracted from the newly flushed needle tissue by Bio S&T (http://www.biost.com/, Montreal, QC, Canada) using the cetyltrimethylammonium bromide method and HMW genomic DNA extraction protocol as detailed in the Chromium Genome Reagent Kits Version 2 User Guide (PN-120229). Assessment of DNA integrity by pulsed-field gel electrophoresis indicated DNA sizes were concentrated at the 20 kbp to 250 kbp range. A total of 60 μg of high-quality purified DNA was sent to Canada's Michael Smith Genome Sciences Centre (https://bcgsc.ca/, Vancouver, BC, Canada) to produce a single library using the 10 × Genomics Chromium system, as previously described (Lo *et al.* 2020). An additional 4 Illumina-compatible libraries, 2 with estimated fragment sizes of 400 bp and 2 with estimated fragment sizes of 800 bp, were constructed. The resulting 10 × Genomics and Illumina-compatible libraries were sequenced on an Illumina HiSeqX instrument yielding 5 lanes of paired-end 150 bp reads and 4 lanes of paired-end 250 bp reads, respectively (Supplementary Table 1 in Supplementary File 1).

### Mitochondrial genome assembly

Given that mitochondrial genome assemblies are available for a number of spruce taxa (Jackman *et al.* 2015, 2020; Sullivan *et al.* 2020; Gagalova *et al.* 2022), the *P. mariana* mitochondrial genome was also assembled here to be used for downstream analyses. Adapter trimming was performed on all reads using Trimadap vr11 (Li 2014), then assembled with ABySS v2.1.0 (Jackman *et al.* 2017) at various *k-*mer sizes (*k* = 64, 72, 80, 88, 96, 104, 116, 122, 128) and *k*-mer multiplicity thresholds (*kc* = 3, 4). The assembly with the highest NG50 (*k* = 116; *kc* = 3) was determined by QUAST v5.0.2 (Gurevich *et al.* 2013) and mitochondrial DNA sequences were extracted based on BWA-MEM alignments of the scaffolds to a reference interior spruce mitochondrial genome (GenBank accessions MK697696–MK697708) (Jackman *et al.* 2015). These sequences were then error-corrected with Tigmint v1.1.2 (Jackman *et al.* 2018), then passed to ARCS v1.0.6 (Yeo *et al.* 2018) and LINKS v1.8.7 (Warren, Yang, *et al.* 2015) (m = 4-20000; k = 20; l = 10; a = 0.1) for scaffolding.

### Genome assembly

Two rounds of read-merging were performed on the adapter-trimmed reads. The first round consisted of cascading Konnector runs (Vandervalk *et al.* 2015), where the 10 × Genomics and Illumina HiSeq reads were merged using *k* values ranging from 115–75 and 235–75, respectively, both with a step size of −10. Reads that were unable to be merged in the first round were subjected to a second round of merging with abyss-mergepairs (Jackman *et al.* 2017). The longer pseudo-reads and any remaining unmerged reads were then assembled using ABySS v2.2.5 (*k* = 96, 112, 128, 144, 160; *kc* = 3, 4). The best assemblies, as assessed by abyss-fac (Jackman *et al.* 2017), were passed to ntJoin v1.0.3 (Coombe *et al.* 2020) to perform iterative assembly-guided scaffolding runs, each with the following parameters: no_cut = True; k = 32; w = 250; reference_weights =’2’. Following scaffolding, a round of misassembly correction was performed on the resulting assembly using Tigmint v1.1.2 with span = 2. In addition to the post-ntJoin assembly, 10 × Genomics Chromium reads were also passed as input. This was followed by another round of scaffolding using ARCS v1.1.1 and LINKS v1.8.6 (c = 3; l = 3; a = 0.9; z = 3000; s = 90). Introduced gaps were filled with Sealer v2.2.3 (Paulino *et al.* 2015) (L = 150; P = 10 l; k = 75,85,95,105,115), yielding the final genome assembly.

### Identification and annotation of protein-coding sequences

Prior to annotating the genome assembly, repeat regions were masked using RepeatMasker v4.1.1 (Chen 2004). A custom *P. mariana* repeat library was constructed using both LTR_retriever v2.9.0 (Ou and Jiang 2018) and RepeatModeler v2.0.1 (Flynn *et al.* 2020), then supplied as input to RepeatMasker to supplement the RepBase v22.08 repeat library (Bao *et al.* 2015). Subsequently, gene models were identified in the repeat-masked genome assembly using BRAKER v2.1.6 (Brůna *et al.* 2021) (--softmasking --etpmode), providing both protein sequences and RNA-seq alignments as evidence as well as an AUGUSTUS model that was pretrained with BUSCO v4.1.4 (Stanke *et al.* 2006, Simão *et al.* 2015) (--long). Specifically, a total of 3,463,432 unique proteins from OrthoDB v10 *Viridiplantae* database (Kriventseva *et al.* 2019), UniProtKB/Swiss-Prot plant entries (The Uniprot Consortium 2019), *P. glauca* manual annotations (Warren, Keeling, *et al.* 2015), and high-quality proteins from annotations found in at least 3 of 4 other spruce taxa (Gagalova *et al.* 2022) selected using a reciprocal best hit (RBH) approach were used (Supplementary Table 2 in Supplementary File 1). Reciprocal BLAST searches were performed between the North American spruce with the most annotations, *P. engelmannii,* and each of the other 3 North American spruces (Gagalova *et al.* 2022). Proteins found in at least 2 of the 3 reciprocal BLAST search results were considered common amongst the North American spruces and included as evidence. To compile the RNA evidence, publicly available *P. mariana* Illumina HiSeq 2 × 100 bp reads derived from seed tissue were obtained (SRA accessions: SRR9595774 and SRR9595777) (Shao *et al.* 2019) and subjected to adapter trimming as well as quality filtering with fastp v0.23.1 (Chen *et al.* 2018). By default, reads with at least 40% of bases having a Phred quality score < 15 were filtered out. Contaminant filtering was performed on the remaining reads with BioBloom tools v2.3.3 (Chu *et al.* 2014), where Bloom filters were created from Aphid, Archaea, Bacteria, Fungi, Protozoa, and Viral reference sequences obtained from RefSeq (O'Leary *et al.* 2016) (Supplementary Table 3 in Supplementary File 1). Reads that did not have hits to any of the Bloom filters, and thus not identified as contaminants, were aligned to the repeat-masked genome assembly using HISAT2 v2.2.0 (Kim *et al.* 2015) with --max-intronlen 1000000.

Following BRAKER annotation, complete protein-coding sequences, defined as those with start and stop codons, containing introns ≥10 bp as identified by GAG v2.0.1 (Geib *et al.* 2018), were functionally annotated using EnTAP v0.10.8 (Hart at al. 2020)—an annotation pipeline that assigns functions based on similarity search hits to user-selected databases as well as EggNOG and InterProScan hits. EnTAP was run in protein mode (--runP) with *P. mariana* provided as the target species and Aphid, Bacteria, and Fungi as contaminant taxa. Similarity searches were performed against the Gymno 1.0 PLAZA database (Proost *et al.* 2009), OrthoDB v10 *Viridiplantae* database, UniprotKB/Swiss-Prot plant entries (The Uniprot Consortium 2019), and Uniref90 database (Suzek *et al.* 2015). In addition to these, the UniProtKB/TrEMBL database (The Uniprot Consortium 2019) was also included for contamination-screening purposes.

Annotations with similarity search and/or ontology hits were assessed for the presence of Pfam domains found in *gag* and *pol* genes. Corresponding annotations were removed on the basis that the internal region of all long-terminal repeat (LTR) retrotransposons consists of these 2 genes and thus, were likely missed by RepeatMasker. Furthermore, as fragmentation of a gene over multiple scaffolds could be common in draft assemblies, fragmented genes were identified and flagged if the following criteria were met: (1) TPM < 1, as determined by SALMON v1.3.0 (Patro *et al.* 2017) and (2) in at least 2 sets of read pairs, the paired-end reads mapped to genes on different scaffolds (Supplementary Table 4 in Supplementary File 1). All 883 flagged fragments were grouped into genes based on shared read pair mappings and the longest fragment of each group was then selected as the representative gene fragment for downstream comparative genomics analyses. The quality of the final assembly and annotation were assessed using abyss-fac and BUSCO v5.2.2 with the OrthoDB v10 embryophyta dataset in protein mode.

### Phylogenetic analyses

Three phylogenetic trees were constructed using *P. mariana* genome sequences and publicly available nuclear, chloroplast, and mitochondrial genome sequences of 5 other spruce taxa [Norway spruce (*P. abies*) v1, white spruce (*P. glauca*) v2, Engelmann spruce (*P. engelmannii*) v1, Sitka spruce (*P. sitchensis*) v1, and the tri-hybrid interior spruce (*P. glauca x engelmannii x sitchensis*) v5], and 2 pines [loblolly pine (*P. taeda*) v2.01 and sugar pine (*P. lambertiana*) v1.5] (Coombe *et al.* 2016; Asaf *et al.* 2018; Lin *et al.* 2019a, *2019*b) (Supplementary Table 5 in Supplementary File 1), using MashTree v1.2.0 (Katz *et al.* 2019) in bootstrap mode: mashtree_bootstrap.pl --reps 100 --numcpus 24 *.fa -- --genomeSize [nuclear: 20000000000, chloroplast: 120000, mitochondrion: 5000000] > *.dnd. The resulting trees were visualized with MEGA11 (Tamura *et al.* 2021) and rooted using the pine species as the outgroup.

### Comparative genomics analyses

The use of different workflows can yield different annotations and thus, varying results in downstream analyses (Venkatraman *et al.* 2021). To account for this, the annotation files for *P. mariana* and the aforementioned taxa (*P. abies* v1, *P. glauca* v2, *P. engelmannii* v1, *P. sitchensis* v1, interior spruce v5, *P. taeda* v2.01, and *P. lambertiana* v1.5) were subjected to the same filtering steps prior to comparative genomics analyses—only complete genes with lengths ≥ 1 kbp and introns ≥ 10 bp were kept. The longest transcript per gene was supplied to OrthoFinder v2.5.4 (Emms and Kelly 2019) and run with default settings to identify phylogenetic hierarchical orthogroups, referred to as orthogroups hereinafter.

From the OrthoFinder results, one can obtain species-specific orthogroups on which further analysis can be performed. Of particular interest were those specific to *P. mariana*. RBH analysis was performed to verify that the orthogroups identified as unique to *P. mariana* did not have any orthologs in other species. MMseqs2 v14.7e284 (Steinegger and Söding 2017) was used to perform reciprocal BLAST searches between *P. mariana* specific transcripts and high-quality transcripts annotated in each of the 4 North American *Picea* taxa (Gagalova *et al.* 2022). Those with RBHs in at least one of the four taxa were removed from analyses pertaining to *P. mariana* specific orthogroups.

### Identifying positively selected protein-coding sequences in *P. mariana*

We used the ratio of nonsynonymous to synonymous substitutions (ω = d*N*/d*S*) to detect signatures of positive selection (Kimura 1977; Kryazhimskiy and Plotkin 2008). Given that synonymous mutations tend to be neutral or nearly neutral, when d*N* > d*S*, natural selection is favoring changes in protein-coding sequences more than neutral expectations and thus, the fixed mutations likely provide a fitness advantage (Kimura 1977). For instance, such changes would be beneficial in stressful environmental conditions, such as those imposed by climate fluctuations (Hoffmann and Hercus 2000).

Detecting signals of positive selection based on pairwise estimations of ω is reputed to be a stringent procedure given that positive selection is only detected if the ω averaged over all sites is >1 (Yang and dos Reis 2011). An alternative and more powerful approach is the use of branch-site tests as it allows variation in ω among branches as well as sites and thus, permits detection of positive selection acting on a few amino acids, which is often the case (Zhang 2005). Given a multiple sequence alignment of genes and a species tree with the branches divided into foreground and background (where the foreground branches are those of interest), the parameters of null and alternative models are estimated. The difference between the 2 models pertains to the predefined foreground branches on which positive selection is allowed. A likelihood ratio test (LRT) is then performed to determine if the alternative model fits significantly better than the null model.

Amongst the 6 spruce and 2 pine taxa, a total of 497 single-copy orthogroups were determined by OrthoFinder and used for this analysis. Primary sequence errors can contribute to misalignments and inaccurate branch length estimates, potentially resulting in overestimated ratios of nonsynonymous to synonymous substitution rates (ω = d*N*/d*S*) and consequently, false detection of positively selected genes (Schneider *et al.* 2009; Di Franco *et al.* 2019). To minimize the effects of such errors, nonhomologous sequences and repeats were trimmed and/or masked in each single-copy orthogroup by PREQUAL v1.02 (Whelan *et al.* 2018)—a prealignment filtering tool. Due to the presence of annotation errors in 4 orthogroups, these could not be processed by PREQUAL and were excluded from the analysis. Multiple sequence alignments were generated for the remaining 493 orthogroups using the codon-aware aligner MACSE v2.06 (Ranwez *et al.* 2018), then provided as input with the previously generated MashTree species topology to CODEML, a program implemented in PAML v4.9j (Yang 2007). To test for positive selection in protein-coding sequences along the *P. mariana* lineage, branch-site models A1 and A were used with the branch leading to *P. mariana* set as the foreground. LRTs were conducted to compare the null and alternative models (at α = 0.05) by first calculating the LRT test statistic, then obtaining *P*-values from the cumulative chi-square distribution function with degrees of freedom, df = 1.

## Results and discussion

### Genome sequencing, assembly, and annotation

Sequencing one library constructed using the 10 × Genomics Chromium system, 2 Illumina-compatible libraries with an estimated fragment size of 400 bp, and another 2 Illumina-compatible libraries with an estimated fragment size of 800 bp yielded 2.2 billion 2 × 150 bp reads, 252 million 2 × 250 bp reads, and 341 million 2 × 250 bp reads, respectively, totaling to a fold coverage of approximately 46× (Supplementary Table 1 in Supplementary File 1). All reads were used in the assembly and initial scaffolding stages.

Various ABySS *k* and *kc* parameter combinations were explored, optimizing for the NG50 length metric and the resulting assemblies were merged with subsequent, iterative ntJoin runs, leveraging the contiguity of each individual assembly. Contiguity evaluation of each assembly indicated that the ABySS assembly with *k* = 176 and *kc* = 3 had one of the highest NG50 values (7.2 kbp) and thus, was used as the target for ntJoin runs. Many of the remaining *kc =* 3 assemblies had relatively high NG50s in comparison to those assembled with *kc =* 4 and thus, were provided as references. The long-range information in linked reads was used for misassembly correction and further scaffolding, ultimately yielding a final assembly consisting of 1,560,767 contigs with a NG50 length of 36.0 kbp and reconstruction of 18.3 Gbp (Table 1).

**Table 1.** Assembly and annotation metrics.

Statistic	Value
Assembly size (Gbp)	18.3
Number of scaffolds (> 500** **bp)	1,549,050
Scaffold N50 length (bp)	45,468
Scaffold NG50 length (bp)	35,958
Repeat content (%)	79.1
LTR elements	49.3
Number of protein-coding sequences	66,332
Number of transcripts	68,738
Mean protein-coding sequence length (bp)	4,165
Mean CDS length (bp)	827
Mean exon length (bp)	256
Mean intron length (bp)	1,612

**Fig. 1.**
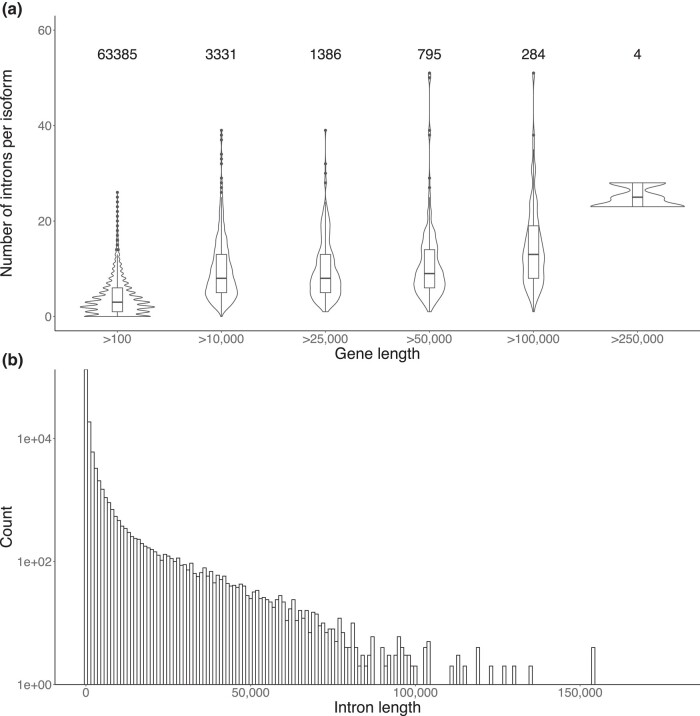
Intron sequences features for all transcript isoforms of each gene annotated in *P. mariana*. a) Number of introns found in each isoform at various gene length thresholds (>100 bp, 10,000 bp, 25,000 bp, 100,000 bp, and 250,000 bp). Each plot is annotated with the number of isoforms that meet the threshold. b) Distribution of intron lengths extracted from all isoforms.

Conifer genomes have a high proportion of repetitive elements, particularly transposable elements (TEs), with the majority of these being LTRs (De La Torre *et al.* 2014). As the presence of these repetitive sequences can lead to false evidence for annotations, it is standard practice to repeat-mask the genome prior to annotation (Yandell and Ence 2012). By supplying a *P. mariana* custom repeat library to RepeatMasker, 79.1% of the genome was identified as repetitive sequences whereas 49.3% consisted of LTR elements (Table 2). This is consistent with findings in other spruce genomes, with reported proportions greater than 70% of highly repetitive DNA (Gagalova *et al.* 2022). The repeat-masked genome was then passed to BRAKER followed by EnTAP for identification of gene models and functional annotation, respectively. A total of 66,332 protein-coding sequences and 68,738 transcripts were annotated. The median number of introns per isoform was 5, with longer genes often yielding transcripts that contain more introns (Fig. 1a). Long introns are characteristic of conifer genomes (De La Torre *et al.* 2014; Stival Sena *et al.* 2014). The longest intron reported had a length of 177 kbp, and 600 introns had lengths greater than 50 kbp (Fig. 1b). Evaluation of annotation quality with BUSCO indicated a total of 416 (25.7%) complete BUSCOs in the annotation.

**Table 2.** Repeat content in *P. mariana*. Proportions of each repeat type were determined using RepeatMasker and LTR_retriever.

Repeat type	Proportion (%)
LTR elements	49.3
** **Gypsy	30.1
** **Copia	12.6
** **Unknown	6.6
LINEs	2.0
DNA transposons	1.0
Simple repeats	0.3
Other repeats	0.3
Unclassified	26.2
**Total repeat content**	79.1

**Fig. 2.**
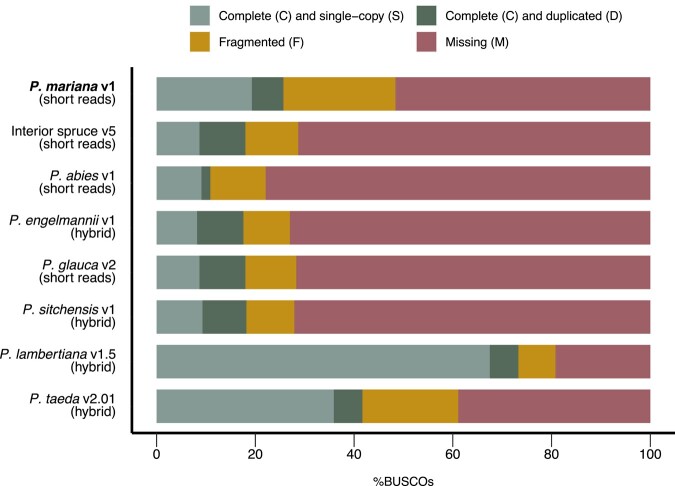
Assessment of *P. mariana* annotation quality (in bold face) relative to other *Picea* and *Pinus* taxa included in downstream analyses. The sequencing read type used in each genome assembly is indicated in parentheses on the y axis, where “hybrid” refers to the use of both short and long reads. Annotation quality was determined using BUSCO v5 in protein mode.

Compared to the published nuclear genome assemblies and annotations of 5 other spruce and 2 pine species (Supplementary Table 5 in Supplementary File 1), the *P. mariana* assembly had a lower contiguity and number of complete BUSCOs than most of them (Supplementary Table 6 in Supplementary File 1). However, this was expected due to the different sequencing technologies and coverage of data that was used for the various assemblies. Whereas the more contiguous genomes were assembled using reads with 80–110 fold coverage and, in some cases, benefitted from the use of long reads (Gonzalez-Ibeas *et al.* 2016; Zimin *et al.* 2017; Gagalova *et al.* 2022), *P. mariana* was assembled with short and linked reads sequenced at 46 × fold coverage. Despite this limited sequencing coverage, the reconstruction of the *P. mariana* genome is consistent with the genome sizes of other spruce species which were reported to be approximately 20 Gbp (Gagalova *et al.* 2022). The numbers of annotated protein-coding sequences and transcripts must be interpreted with caution when compared with the number of genes identified in previous spruce genome sequence studies given the different annotation pipelines and downstream filtering methods used to obtain the final annotations. For instance, BRAKER was used for predicting gene models in *P. mariana*, whereas a combination of AUGUSTUS and EuGene were used for *P. abies* and the MAKER-P pipeline was used for the other North American spruce species (Nystedt *et al.* 2013; Warren, Keeling, *et al.* 2015; Gagalova *et al.* 2022). Furthermore, the presence of frequent long introns interspacing exons in spruce genes (Nystedt *et al.* 2013; Stival Sena *et al.* 2014) coupled with the lower NG50 in the present study compared to Gagalova *et al.* (2022), can lead to the annotation of partial protein-coding sequences. Nonetheless, the higher number of annotated protein-coding sequences, despite applying filters such as completeness (presence of start and stop codon) and minimum intron length of 10 bp (Supplementary Table 4 in Supplementary File 1), was matched by a higher number of complete BUSCOs (Fig. 2, Supplementary Table 6 in Supplementary File 1). This result is not unexpected given the use of BRAKER, a more recently developed annotation pipeline that has been shown to yield annotations with higher BUSCO completeness scores, sensitivity, and precision compared to MAKER (Vuruputoor *et al.* 2023).

### Phylogenetic analyses

**Fig. 3.**
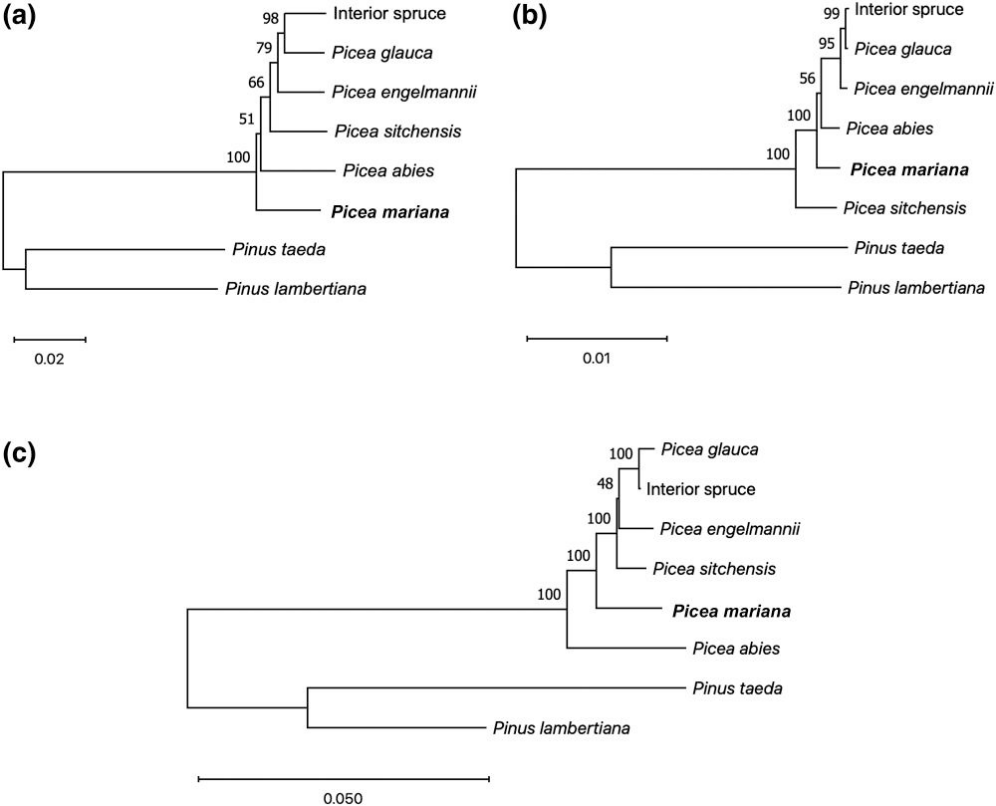
MashTree phylogenies from the a) nuclear, b) chloroplast, and c) mitochondrial genomes of *P. mariana* (in bold face) and related species. MashTree estimates distances based on *k-*mer sketches of the genomes, then constructs phylogenetic trees using the neighbor-joining method. For all 3 phylogenies, the trees were rooted using pine species as outgroups. Branch lengths are proportional to the number of substitutions per nucleotide position. Values at the nodes indicate bootstrap values.

To gain insights on the evolutionary relationships of *P. mariana* in relation to other spruce species, phylogenetic analysis was performed with 6 spruce and 2 pine species for which complete genome sequences were available (Supplementary Table 5 in Supplementary File 1), using pines as outgroups for these analyses. Phylogenetic trees were also assembled using chloroplast and mitochondrial genome sequences, given their uniparental mode of inheritance in Pinaceae (paternal for the chloroplast genome and maternal for the mitochondrial genome) and different tree topologies as observed in previous studies based on specific chloroplast and mitochondrial regions (Bouillé *et al.* 2011; Ran *et al.* 2015; Gagalova *et al.* 2022). The use of chloroplast, mitochondrial, and biparentally inherited nuclear genomes should thus provide a more complete picture of evolutionary relationships among these 6 spruce species. In the nuclear phylogeny, *P. mariana* together with the European *P. abies*, are sister groups to the other North American spruces included in this analysis, namely, *P. glauca*, *P. engelmannii*, *P. sitchensi*s and the hybrid interior spruce (Fig. 3a). Whereas *P. glauca, P. engelmannii*, and *P. sitchensis* frequently hybridize with each other, giving rise to interior spruce (Gagalova *et al.* 2022), attempted crosses between these taxa and *P. mariana* have either failed or had low success rate, indicating higher evolutionary divergence of *P. mariana* relative to the others (Ran *et al.* 2006). However, the position of *P. mariana* relative to *P. abies* remained ambiguous given the weak support of *P. abies* as a sister group to *P. glauca, P. engelmannii*, *P. sitchensis*, and interior spruce.

A similar phylogenetic tree topology was obtained with the chloroplast and mitochondrial genomes, but the positions of *P. sitchensis* and *P. abies* conflicted among phylogenies (Fig. 3). In the chloroplast phylogeny (Fig. 3b), the position of European *P. abies* as a sister group to North American *P. glauca, P. engelmannii*, *P. sitchensis*, and interior spruce was weakly supported. However, in the mitochondrial phylogeny (Fig. 3c), there was strong support for *P. mariana* as a sister group to these other North American species. Furthermore, in the mitochondrial phylogeny and contrary to the chloroplast phylogeny, there was strong support for *P. sitchensis* as a sister group to *P. glauca*, *P. engelmannii*, and interior spruce, with the latter confirmed as a natural tri-hybrid of the former 3 species (Gagalova *et al.* 2022). This is indicative of incomplete reproductive isolation and reduced interspecific genetic divergence among them. The topology of the chloroplast phylogeny placed *P. sitchensis* as an outgroup to all other spruce taxa, which conflicts with the 2 other phylogenies and biogeography. This odd positioning had previously been observed and proposed to result from ancient reticulate evolution affecting the *P. sitchensis* chloroplast genome (Bouillé *et al.* 2011; Sullivan *et al.* 2017; Gagalova *et al.* 2022).

Though weakly supported, the odd positioning of the European *P. abies* as a sister group to *P. glauca* and *P. engelmannii* on the chloroplast phylogeny, and to *P. glauca*, *P. engelmannii*, and *P. sitchensis* on the nuclear phylogenetic tree, might also be indicative of reticulation between *P. abies* and *P. glauca* through ancient gene flow (Chen *et al.* 2010). Such contact could date back to an interglacial period approximately 400,000 years ago when spruce species were dominant in Greenland, in particular *P. abies* (de Vernal and Hillaire-Marcel 2008). Such a tree topology was not observed on the mitochondrial phylogeny where a strong geographic structure placing *P. mariana* as the sister group to all other North American spruces was observed and expected from ancient geographical speciation (Bouillé *et al.* 2011). Indeed, spruce mitochondrial genomes are dispersed by spruce seeds only, which disseminate across much smaller distances than pollen and thus, chloroplast genomes. As such, the mitochondrial tree may be more indicative of vertical descent associated with phylogeographic speciation, while the chloroplast tree would integrate horizontal transfers by pollen though more or less ancient reticulate evolution between already genetically distinct lineages (Gérardi *et al.* 2010; Bouillé *et al.* 2011; Sullivan *et al.* 2017; Gagalova *et al.* 2022). As for the nuclear phylogeny, given the biparental inheritance of the nuclear genome, it well reflects a blend of both types of evolution.

### Comparative genomics

In a recent study, Gagalova *et al.* (2022) showed that the allopatric and ecologically divergent *P. glauca* and *P. sitchensis* had mostly distinct sets of rapidly evolving genes under positive selection, largely related to stress and stimuli response. Although the natural ranges of *P. mariana* and *P. glauca* are mostly sympatric, the 2 species have also adapted to distinct climatic and ecological niches. Black spruce is often found on cold, wet, nutrient-poor soils, whereas white spruce tends to inhabit sites with warmer, well-drained soils (Nicklen *et al.* 2021). As several studies have associated species-specific genes with environmental stress responses and unique traits (Li *et al.* 2009; Arendsee *et al.* 2014), further delineating and investigating protein-coding sequences found specific to *P. mariana* may provide insights on how it has adapted to its rather specific ecological niche among the spruce species analyzed here.

To compare the protein-coding sequence content among the 6 spruce and 2 pine species, an orthogroup analysis was performed using OrthoFinder. In the context of the present taxa sampling, this analysis resulted in the identification of 34,776 orthogroups in total, with 2,869 being shared amongst all species and 560 found specific to *P. mariana* (Supplementary Table 7 in Supplementary File 1).

### Analysis of *P. mariana* specific orthogroups

**Fig. 4.**
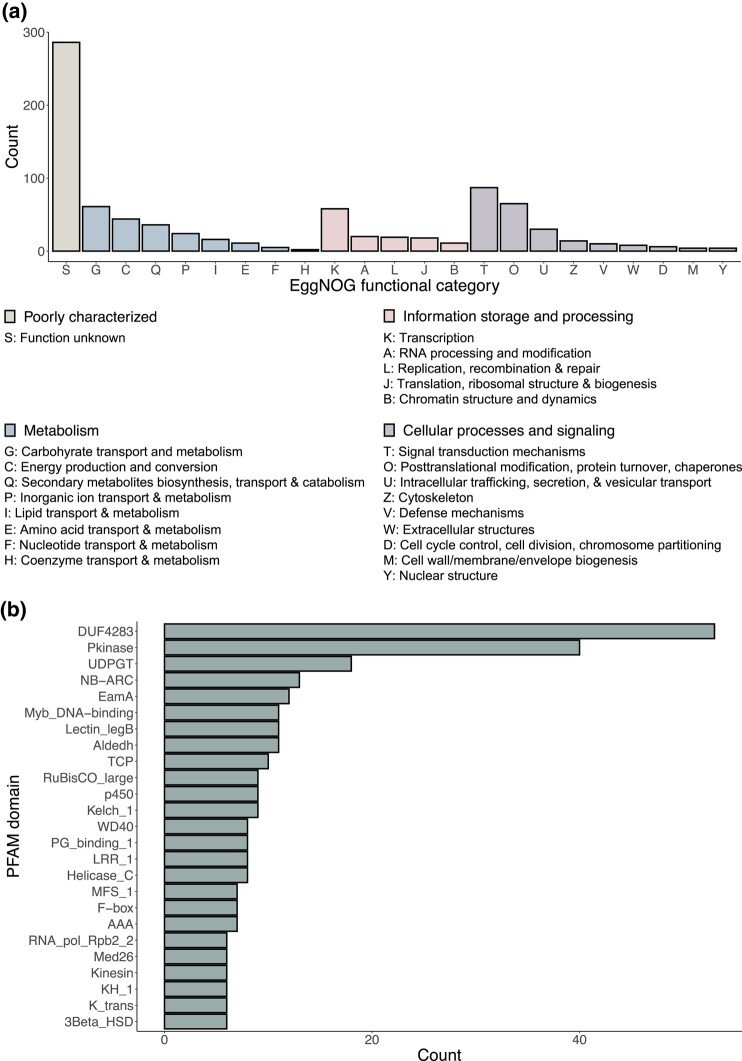
Distribution of a) EggNOG functional categories and b) best matching PFAM domains (n > 5). Functional category annotations were assigned based on hits to entries in the EggNOG database, while PFAM annotations were assigned via hits in EggNOG and/or InterPro databases as part of the EnTAP annotation pipeline. If a protein-coding sequence was assigned to multiple functional categories, each category was counted separately.

Of the 1,900 protein-coding sequences assigned to *P. mariana* specific orthogroups, 109 sequences had RBHs with genes in at least one of the four North American spruces and thus, were removed from subsequent analyses. On further analysis of the remaining *P. mariana* specific protein-coding sequences, 792 were assigned functional categories based on orthologous hits in the EggNOG database. Majority of the predicted functions were unknown (Fig. 4a), with many protein-coding sequences containing a domain of unknown function (DUF4283); however, the EamA and F-box domains were also highly prevalent (Fig. 4b, Supplementary Fig. 1 in Supplementary File 1) and these gene families have been partially characterized in *Viridiplantae*. The EamA gene family is involved in transport (Västermark *et al.* 2011) and sequences belonging to the F-box superfamily are responsible for controlling diverse biological processes including growth, development, and abiotic stress tolerance (Abd-Hamid *et al.* 2020; *Rao and Virupapuram 2021*).

Apart from those classified as function unknown, there was a relatively large presence of sequences in the following categories: signal transduction mechanisms; post-translational modification, protein turnover, chaperones; transcription; carbohydrate transport and metabolism; secondary metabolites biosynthesis, transport, and catabolism (Fig. 4a). Among those classified under signal transduction mechanisms, Pkinase domains were particularly prevalent (Fig. 4b, Supplementary Fig. 1 in Supplementary File 1). NB-ARC and LRR 1 domains, both found in NLR proteins which are widely known to play a central role in resilience to biotic and abiotic stresses (Van Ghelder *et al.* 2019; Ence *et al.* 2022), were present in high numbers as well. Interestingly, a study aimed at developing a repertoire of conifer NLR genes identified more in *P. mariana* compared to *P. glauca* (Van Ghelder *et al.* 2019), thereby supporting our finding of numerous NB-ARC and LRR 1 domain-containing sequences unique to *P. mariana*. Additionally, Lectin LegB domains were often found in sequences associated with signal transduction mechanisms. Various studies have demonstrated the critical role that this domain plays in pathogen response (Singh and Zimmerli 2013; Lannoo and Van Damme 2014) and a few identifying links to abiotic stress response and developmental processes (Wan *et al.* 2008; Li *et al.* 2014; Zhao *et al.* 2019).

Domains found in sequences annotated with the post-translational modification, protein turnover, chaperones EggNOG functional category included PG binding 1 and AAA (Fig. 4b, Supplementary Fig. 1 in Supplementary File 1). Many sequences containing the PG binding 1 domain were annotated with an InterPro domain in matrix metalloproteinases (MMPs)—Pept M10 metallopeptidase. Few plant MMPs have been characterized in detail, but a recent study assessing genetic variation among drought resilient Norway spruce trees identified a SNP in a MMP family gene that had significant association with wood density (*R*^2^ = 0.25–0.26) (Trujillo-Moya *et al.* 2018).

Myb Dna-binding domain was the most frequent domain in the transcription EggNOG functional category (Fig. 4b, Supplementary Fig. 1 in Supplementary File 1), accounting for 11 of the 58 sequences assigned to that category. Previously, several R2R3-MYB genes had been identified and characterized in *P. mariana* and *P. glauca* (Xue *et al.* 2003; Bedon *et al.* 2007). However, given the large size of the R2R3-MYB family, with over 120 genes reported in angiosperms (Riechmann *et al.* 2000), it is likely that those previously identified make up only a fraction of the R3R3-MYB clade in spruce, let alone those that have yet to be identified in other MYB clades. Following Myb Dna-binding, the Med26 domain, which plays a possible role in transcription elongation (Mathur *et al.* 2011), and RNA pol Rpb2 2, the domain characteristic of genes encoding the second-largest subunit of DNA-dependent RNA polymerase II, also appeared frequently in the transcription EggNOG functional category.

Protein-coding sequences related to carbohydrate transport and metabolism were often categorized under energy production and conversion. Within these 2 EggNOG functional categories, UDPGT, Alde H, RuBisCO large, and MFS 1 domains were the most represented (Fig. 4b, Supplementary Fig. 1 in Supplementary File 1). The p450 domain was most prevalent in the secondary metabolite biosynthesis, transport, and catabolism EggNOG functional category. These domains are characteristic of cytochrome P450 oxygenases (CYPs) known to play important roles in oleoresin defenses of conifers and in interactions of conifers with insect pests and pathogens (Celedon and Bohlmann 2019; Chiu and Bohlmann 2022). Expansions within CYP subfamilies have been detected (Warren, Keeling, *et al.* 2015); therefore, the p450 domain-containing *P. mariana* specific protein-coding sequences may be members of the CYP gene family that have not been annotated in the species included in this analysis.

In all, 999 *P. mariana* specific protein-coding sequences were not assigned functional categories as there were no orthologous hits in the EggNOG database, but 122 of these sequences were annotated with PFAM domains based on InterPro hits. The most prevalent PFAM domain amongst these was the TCP domain, which has been found in transcription factors regulating plant growth and development (Cubas *et al.* 1999).

There are various potential factors that could contribute to the presence of these *P. mariana* specific protein-coding sequences and additional studies are needed to better understand their evolution. Black spruce exhibits unique ecological traits such as being adapted to the large variety of soil types and moisture conditions found in the temperate and boreal forests (Nicklen *et al.* 2021), as well as being able to maintain itself vegetatively for centuries in the inhospitable growing conditions of the northern open boreal forest and subarctic tundra through layering (Laberge *et al.* 2000). Thus, it would be interesting to investigate whether these coding sequences are encoded by novel genes related to these traits. It is also possible that some sequences appear species-specific due to sequence divergence leading to failed inference of orthologs and/or unannotated sequences in the other spruce and pine species. For instance, within the MYB gene family, the amino acid sequences outside of the MYB DNA-binding domains are highly divergent (Bedon *et al.* 2010), which may be challenging for multiple sequence alignment tools and thus, complicate the process of gene tree and orthogroup inference. Furthermore, several highly abundant PFAM domains in *P. mariana* specific protein-coding sequences were also found to be abundant in unannotated *P. lambertiana* transcripts, namely DUF4283, PKinase, NB-ARC, WD40, and LRR 1 (Gonzalez-Ibeas *et al.* 2016).

### Protein-coding sequences under positive selection

**Fig. 5.**
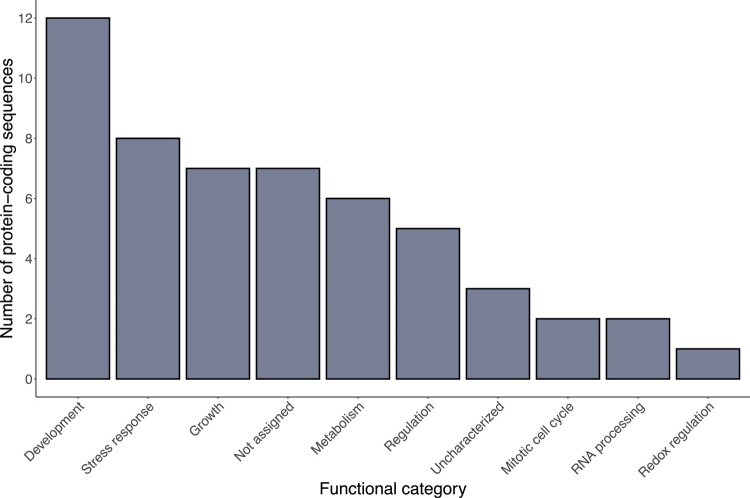
Functional classes of the 45 positively selected protein-coding sequences. In total, 38 sequences had a match with an *Arabidopsis* protein from the Araport11 database (blastp evalue < e−20) and could be associated to a functional class based on homology searches and/or literature searches. If a protein-coding sequence was assigned to multiple functional classes, each class was counted separately. “Not assigned” refers to sequences without hits in the Araport11 database, whereas “uncharacterized” refers to those that had matches but the functions of those *Arabidopsis* proteins have not been fully characterized yet.

To identify positively selected protein-coding sequences in *P. mariana*, branch-site tests were conducted on the 497 single-copy orthogroups identified between all 8 conifer taxa considered in this study. Of these, 45 (9.1%) were identified as positively selected (Supplementary Table 8), 38 of which could be annotated with one or more functional categories based on matches with known proteins in the Araport11 database (Cheng *et al.* 2017) and/or literature search highlighting a diverse set of molecular functions (Supplementary Table 9). The most represented functional category was development (12 sequences), followed by response to stress (8 sequences) and growth (7 sequences) (Fig. 5). Among them, several protein-coding sequences appear essential either for plant survival or plant adaptation to environmental conditions as illustrated by the following examples.

Twelve protein-coding sequences were found to be involved in development, 7 of which were only assigned to that category. These include 2 sequences whose homologs regulate flowering time in *Arabidopsis*: the developmental protein FRIGIDA (Michaels *et al.* 2004), also involved in temperature adaptation (Tabas-Madrid *et al.* 2018), and the serine/threonine-protein kinase WNK1 that regulates flowering time by modulating the photoperiod pathway (Wang *et al.* 2008). Furthermore, there was an aspartate/glutamate/uridylate kinase family protein that has been identified to be a trihelix/aa-kinase chimera associated with leaf development (Kuromori *et al.* 2006; Kaplan-Levy *et al.* 2012). The other 4 sequences included an ARID domain-containing protein which is a seed-specific transcription factor in *Arabidopsis* (Zheng *et al.* 2014), a NAC transcription factor involved in xylem formation (Endo *et al.* 2015), ARF19 which is responsible for regulating various auxin-mediated development processes including lateral root formation (Okushima *et al.* 2005, 2007) and TMKL1, whose function has yet to be fully characterized but has been suggested to mediate vascular tissue development (Wu *et al.* 2016).

The remaining 5 of 12 sequences involved in development were also associated with other functional classes. One protein-coding sequence plays a role in RNA processing as well as development—RRD1 participates in mitochondrial mRNA deadenylation, which is fundamental in controlling early lateral root organogenesis (Otsuka *et al.* 2021). There were also 2 protein-coding sequences involved in both growth and development. One is an ankyrin repeat-containing protein essential for chloroplast biogenesis (Shen *et al.* 2010), while the other is a member of the IQ67-domain (IQD) protein family known to regulate plant growth, lateral organ polarity, and basal defense response against environmental cues (Bürstenbinder *et al.* 2017; Barda and Levy 2022). The last 2 sequences are involved in stress response and development: a development and cell death (DCD) domain-containing protein (Tenhaken *et al.* 2005) and AAC2, which encodes a mitochondrial ADP/ATP carrier that has been suggested to play a role in the mechanisms of ABA-mediated stress response (Kharenko *et al.* 2011).

A total of 8 sequences encoded proteins related to stress response. Three are involved in plant immunity or plant immunity regulation: a member of the pleiotropic drug resistance family, the PBL27 serine–threonine kinase involved in a cascade that leads to chitin-induced immunity (Kawasaki *et al.* 2017) and a homolog of the AtWRKY3 transcription factor regulating the expression of the pathogen induced gene NPR1 (Yu *et al.* 2001). PLIP2 is a glycerolipid A1 lipase that provides tolerance to various abiotic stresses, including cold, by ABA-mediated synthesis of jasmonate and oxolipins (Wang *et al.* 2018). Four of the 8 stress-related protein-coding sequences were assigned to another functional category along with stress response, 2 of which have been mentioned previously—a DCD domain-containing protein and AAC2, both contributing to plant development as well. Besides those, a sequence encoding DGD1 was additionally annotated with metabolism as it is responsible for the synthesis of DGDG, a glycolipid critical for the stabilization of chloroplast membranes, thereby conferring thermotolerance (Kobayashi *et al.* 2014). APUM8 was also annotated with RNA-processing and has been implicated in salt stress tolerance (Huang *et al.* 2018).

Five protein-coding sequences had direct or indirect roles in growth, cell elongation, and plant height in *Arabidopsis*: a filament-like protein (Chen *et al.* 2016), an actin-binding FH2 (formin 2) family protein (Vidali *et al.* 2009), DRACULA2 (Gallemí *et al.* 2016), CNGC8 which has been shown to be essential for pollen tube tip growth (Tunc-Ozdemir *et al.* 2013), and KORRIGAN2 (Mølhøj *et al.* 2001) whose homolog is also associated to early growth in pine (Cabezas *et al.* 2015).

The functional categories of the high d*N*/d*S* protein-coding sequences found in the present study overlap well with those reported by Buschiazzo *et al.* (2012) and De La Torre *et al.* (2015), both of which used estimations of d*N*/d*S* to detect positive selection. Eight gene families implicating high d*N*/d*S* values were found in common between the present study and that of De La Torre *et al.* (2015), including auxin responsive protein, eukaryotic aspartyl protease, alkaline and neutral invertase, ABC transporter, cyclic nucleotide gated channel, FRIGIDA-like proteins, ankyrin repeat-containing protein, and the O-Glycosyl hydrolases family 17. However, the specific protein-coding sequences with high d*N*/d*S* values were different between studies, including the fact that here, we considered only single-copy genes in the d*N*/d*S* analysis. These studies should be viewed as complementary, 2 being based on transcriptome data and our study on genomic data. Furthermore, there was a large overlap between the abundant functional categories found in this study and those presented in Gagalova *et al.* (2022), where the most rapidly evolving genes shared by *P. sitchensis* and *P. glauca* were identified by estimating the ratios of nonsynonymous to synonymous SNPs (SNP A/S) for each species.

## Conclusions

We produced the first nuclear genome assembly of *P. mariana* with a NG50 length of 36.0 kbp and reconstruction of 18.3 Gbp. This is comparable to the estimated haploid genome size reported to be around 17.5 Gbp based on the measured C-value of 17.4 pg (Bai *et al.* 2012; Mann *et al.* 2013). This estimate indicates that the large size of the spruce nuclear genome is a feature shared by multiple lineages. Hence, given the average divergence time in the scale of 10 to 20 Myr between major spruce lineages leading to *P. mariana*, *P. abies*, and *P. glauca* (Bouillé and Bousquet 2005), our results support the finding that much of the spruce genome expansion would thus well predate this era and be shared by common ancestry (Nystedt *et al.* 2013).

Substantial genetic differentiation was detected between the black spruce nuclear and organellar genomes and those of other North American spruce species, reflecting the reproductive isolation of black spruce from the others and the more ancient divergence of its lineage. At the same time, signatures of at least 2 occurrences of ancient reticulate evolution of the spruce chloroplast and nuclear genomes were detected. These support evidence of incomplete lineage sorting (Bouillé and Bousquet 2005) and slow speciation characterized by delayed establishment of reproductive isolation influenced by the large effective population sizes and airborne dispersion of pollen over potentially long distances in these species (Bousquet *et al.* 2021).

Analysis of specific biological functions and positive selection revealed an abundance of genes implicated in development and stress responses, whether for biotic or abiotic factors. This is congruent with the often-harsh environments or climates characterizing the ecological niches occupied by spruce species, particularly black spruce as it is found mostly in the boreal forests of North America. This pattern is largely shared with observations from previous studies of molecular adaptation in spruces (e.g. Hornoy *et al.* 2015; Yeaman *et al.* 2016; Depardieu *et al.* 2021; Gagalova *et al.* 2022), although taxon-specific genes under positive selection appear much more frequent than shared ones, at least between spruces (Gagalova *et al.* 2022). This is likely related to the existence of large gene families and functional redundancy potentially leading to subfunctionalization or neofunctionalization (Warren, Keeling, *et al.* 2015; Stival Sena *et al.* 2018; Van Ghelder *et al.* 2019; De La Torre *et al.* 2020), which is likely beneficial in developing multiple molecular strategies for stimuli and stress response.

With black spruce predicted to decline due to increasing temperature across its natural range (Thomson *et al.* 2009), and the increasing frequency of extreme weather events such as late cold spells and drought episodes under mid-northern latitudes (Benomar *et al.* 2022; Laverdière *et al.* 2022), much remains to be understood about the specific mechanisms and molecular basis of genetic adaptation in this ecologically and economically important species. We expect that the assembly and annotation of the black spruce nuclear genome will aid the forest research community in gaining a better understanding of the different mechanisms and capacities for local adaptation to climate warming and increasing instability, thus contributing to the development of molecular-assisted breeding strategies and guidelines to orientate assisted migration.

## Supplementary Material

jkad247_Supplementary_Data

## Data Availability

Raw sequencing reads are available on NCBI under BioProject PRJNA610902. This Whole Genome Shotgun project has been deposited at DDBJ/ENA/GenBank under the accession JASDQU000000000. The version described in this article is version JASDQU010000000. The genome annotation is available on Zenodo (Lo *et al.* 2023). The mitochondrial genome assemblies for *P. mariana* (Marr *et al.* 2023), *P. engelmannii* (Coombe *et al.* 2023a), and *P. glauca* (Coombe *et al.* 2023b) are also available on Zenodo. Supplemental material available at G3 online.

## References

[jkad247-B1] Abd-Hamid N-A , Ahmad-Fauzi M-I, Zainal Z, Ismail I. 2020. Diverse and dynamic roles of F-box proteins in plant biology. Planta. 251(3):68. doi:10.1007/s00425-020-03356-8. 32072251

[jkad247-B2] Aitken SN , Yeaman S, Holliday JA, Wang T, Curtis-McLane S. 2008. Adaptation, migration or extirpation: climate change outcomes for tree populations. Evol Appl. 1(1):95–111. doi:10.1111/j.1752-4571.2007.00013.x. 25567494 PMC3352395

[jkad247-B3] Allen CD , Breshears DD, McDowell NG. 2015. On underestimation of global vulnerability to tree mortality and forest die-off from hotter drought in the Anthropocene. Ecosphere. 6(8):art129. doi:10.1890/ES15-00203.1.

[jkad247-B4] Allen CD , Macalady AK, Chenchouni H, Bachelet D, McDowell N, Vennetier M, Kitzberger T, Rigling A, Breshears DD, Hogg EH, et al 2010. A global overview of drought and heat-induced tree mortality reveals emerging climate change risks for forests. For Ecol Manag. 259(4):660–684. doi:10.1016/j.foreco.2009.09.001.

[jkad247-B5] Arendsee ZW , Li L, Wurtele ES. 2014. Coming of age: orphan genes in plants. Trends Plant Sci. 19(11):698–708. doi:10.1016/j.tplants.2014.07.003. 25151064

[jkad247-B6] Asaf S , Khan AL, Khan MA, Shahzad R, Lubna KS, Al-Harrasi A, Al-Rawahi A, Lee I-J. 2018. Complete chloroplast genome sequence and comparative analysis of loblolly pine (*Pinus taeda* L.) with related species. PLoS One. 13(3):e0192966. doi:10.1371/journal.pone.0192966. 29596414 PMC5875761

[jkad247-B7] Bai C , Alverson WS, Follansbee A, Waller DM. 2012. New reports of nuclear DNA content for 407 vascular plant taxa from the United States. Ann Bot. 110(8):1623–1629. doi:10.1093/aob/mcs222. 23100602 PMC3503501

[jkad247-B8] Bao W , Kojima KK, Kohany O. 2015. Repbase update, a database of repetitive elements in eukaryotic genomes. Mob DNA. 6(1):11. doi:10.1186/s13100-015-0041-9. 26045719 PMC4455052

[jkad247-B9] Barda O , Levy M. 2022. IQD1 Involvement in hormonal signaling and general defense responses against *Botrytis cinerea*. Front Plant Sci. 13:845140. doi:10.3389/fpls.2022.845140. 35557724 PMC9087847

[jkad247-B10] Beaulieu J , Perron M, Bousquet J. 2004. Multivariate patterns of adaptive genetic variation and seed source transfer in *Picea mariana*. Can J For Res. 34(3):531–545. doi:10.1139/x03-224.

[jkad247-B11] Bedon F , Bomal C, Caron S, Levasseur C, Boyle B, Mansfield SD, Schmidt A, Gershenzon J, Grima-Pettenati J, Séguin A, et al 2010. Subgroup 4 R2R3-MYBs in conifer trees: gene family expansion and contribution to the isoprenoid- and flavonoid-oriented responses. J Exp Bot. 61(14):3847–3864. doi:10.1093/jxb/erq196. 20732878 PMC2935864

[jkad247-B12] Bedon F , Grima-Pettenati J, Mackay J. 2007. Conifer R2R3-MYB transcription factors: sequence analyses and gene expression in wood-forming tissues of white spruce (*Picea glauca*). BMC Plant Biol. 7(1):17. doi:10.1186/1471-2229-7-17. 17397551 PMC1851958

[jkad247-B13] Benomar L , Bousquet J, Perron M, Beaulieu J, Lamara M. 2022. Tree maladaptation under mid-latitude early spring warming and late cold spell: implications for assisted migration. Front Plant Sci. 13:920852. doi:10.3389/fpls.2022.920852. 35874013 PMC9298535

[jkad247-B14] Birol I , Raymond A, Jackman SD, Pleasance S, Coope R, Taylor GA, Yuen MMS, Keeling CI, Brand D, Vandervalk BP, et al 2013. Assembling the 20 Gb white spruce (*Picea glauca*) genome from whole-genome shotgun sequencing data. Bioinformatics. 29(12):1492–1497. doi:10.1093/bioinformatics/btt178. 23698863 PMC3673215

[jkad247-B15] Bouillé M , Bousquet J. 2005. Trans-species shared polymorphisms at orthologous nuclear gene loci among distant species in the conifer *Picea* (Pinaceae): implications for the long-term maintenance of genetic diversity in trees. Am J Bot. 92(1):63–73. doi:10.3732/ajb.92.1.63. 21652385

[jkad247-B16] Bouillé M , Senneville S, Bousquet J. 2011. Discordant mtDNA and cpDNA phylogenies indicate geographic speciation and reticulation as driving factors for the diversification of the genus *Picea*. Tree Genet Genomes. 7(3):469–484. doi:10.1007/s11295-010-0349-z.

[jkad247-B17] Bousquet J , Gérardi S, de Lafontaine G, Jaramillo-Correa JP, Pavy N, Prunier J, Lenz P, Beaulieu J. 2021. Spruce population genomics. In: Raja OP, editors. Population Genomics. Cham: Springer International Publishing. p. 64p.

[jkad247-B18] Brůna T , Hoff KJ, Lomsadze A, Stanke M, Borodovsky M. 2021. BRAKER2: automatic eukaryotic genome annotation with GeneMark-EP+ and AUGUSTUS supported by a protein database. NAR Genom Bioinform. 3(1):lqaa108. doi:10.1093/nargab/lqaa108. 33575650 PMC7787252

[jkad247-B19] Bürstenbinder K , Mitra D, Quegwer J. 2017. Functions of IQD proteins as hubs in cellular calcium and auxin signaling: a toolbox for shape formation and tissue-specification in plants? Plant Signal Behav. 12(6):e1331198. doi:10.1080/15592324.2017.1331198. 28534650 PMC5566250

[jkad247-B20] Buschiazzo E , Ritland C, Bohlmann J, Ritland K. 2012. Slow but not low: genomic comparisons reveal slower evolutionary rate and higher dN/dS in conifers compared to angiosperms. BMC Evol Biol. 12(1):8. doi:10.1186/1471-2148-12-8. 22264329 PMC3328258

[jkad247-B21] Cabezas JA , González-Martínez SC, Collada C, Guevara MA, Boury C, de María N, Eveno E, Aranda I, Garnier-Géré PH, Brach J, et al 2015. Nucleotide polymorphisms in a pine ortholog of the *Arabidopsis* degrading enzyme cellulase KORRIGAN are associated with early growth performance in *Pinus pinaster*. Tree Physiol. 35(9):1000–1006. doi:10.1093/treephys/tpv050. 26093373

[jkad247-B22] Celedon JM , Bohlmann J. 2019. Oleoresin defenses in conifers: chemical diversity, terpene synthases and limitations of oleoresin defense under climate change. New Phytol. 224(4):1444–1463. doi:10.1111/nph.15984. 31179548

[jkad247-B24] Chen J , Källman T, Gyllenstrand N, Lascoux M. 2010. New insights on the speciation history and nucleotide diversity of three boreal spruce species and a tertiary relict. Heredity (Edinb). 104(1):3–14. doi:10.1038/hdy.2009.88. 19639012

[jkad247-B25] Chen J , Källman T, Ma X, Gyllenstrand N, Zaina G, Morgante M, Bousquet J, Eckert A, Wegrzyn J, Neale D, et al 2012. Disentangling the roles of history and local selection in shaping clinal variation of allele frequencies and gene expression in Norway spruce (*Picea abies*). Genetics. 191(3):865–881. doi:10.1534/genetics.112.140749. 22542968 PMC3389980

[jkad247-B26] Chen L , Peng Y, Tian J, Wang X, Kong Z, Mao T, Yuan M, Li Y. 2016. TCS1, A microtubule-binding protein, interacts with KCBP/ZWICHEL to regulate trichome cell shape in *Arabidopsis thaliana*. PLoS Genet. 12(10):e1006266. doi:10.1371/journal.pgen.1006266. 27768706 PMC5074588

[jkad247-B23] Chen N . 2004. Using RepeatMasker to identify repetitive elements in genomic sequences. Curr Protoc Bioinformatics. Chapter 4:Unit 4.10. doi:10.1002/0471250953.bi0410s05. 18428725

[jkad247-B27] Chen S , Zhou Y, Chen Y, Gu J. 2018. Fastp: an ultra-fast all-in-one FASTQ preprocessor. Bioinformatics. 34(17):i884–i890. doi:10.1093/bioinformatics/bty560. 30423086 PMC6129281

[jkad247-B28] Cheng C-Y , Krishnakumar V, Chan AP, Thibaud-Nissen F, Schobel S, Town CD. 2017. Araport11: a complete reannotation of the *Arabidopsis thaliana* reference genome. Plant J. 89(4):789–804. doi:10.1111/tpj.13415. 27862469

[jkad247-B29] Chiu CC , Bohlmann J. 2022. Mountain pine beetle epidemic: an interplay of terpenoids in host defense and insect pheromones. Annu Rev Plant Biol. 73(1):475–494. doi:10.1146/annurev-arplant-070921-103617. 35130442

[jkad247-B30] Chu J , Sadeghi S, Raymond A, Jackman SD, Nip KM, Mar R, Mohamadi H, Butterfield YS, Robertson AG, Birol I. 2014. Biobloom tools: fast, accurate and memory-efficient host species sequence screening using bloom filters. Bioinformatics. 30(23):3402–3404. doi:10.1093/bioinformatics/btu558. 25143290 PMC4816029

[jkad247-B31] Coombe L , Nikoli V, Chu J, Birol I, Warren RL. 2020. Ntjoin: fast and lightweight assembly-guided scaffolding using minimizer graphs. Bioinformatics. 36(12):3885–3887. doi:10.1093/bioinformatics/btaa253. 32311025 PMC7320612

[jkad247-B32] Coombe L , Warren RL, Birol I. 2023a. Picea engelmannii isolate Se404–851 mitochondrial genome assembly. Zenodo. doi:10.5281/zenodo.7828263.

[jkad247-B33] Coombe L , Warren RL, Birol I. 2023b. Picea glauca isolate WS77111 mitochondrial genome assembly. Zenodo. doi:10.5281/zenodo.7828292.

[jkad247-B34] Coombe L , Warren RL, Jackman SD, Yang C, Vandervalk BP, Moore RA, Pleasance S, Coope RJ, Bohlmann J, Holt RA, et al 2016. Assembly of the complete Sitka spruce chloroplast genome using 10X genomics’ GemCode sequencing data. PLoS One. 11(9):e0163059. doi:10.1371/journal.pone.0163059. 27632164 PMC5025161

[jkad247-B35] Cubas P , Lauter N, Doebley J, Coen E. 1999. The TCP domain: a motif found in proteins regulating plant growth and development. Plant J. 18(2):215–222. doi:10.1046/j.1365-313x.1999.00444.x. 10363373

[jkad247-B36] De La Torre AR , Birol I, Bousquet J, Ingvarsson PK, Jansson S, Jones SJM, Keeling CI, MacKay J, Nilsson O, Ritland K, et al 2014. Insights into conifer giga-genomes. Plant Physiol. 166(4):1724–1732. doi:10.1104/pp.114.248708. 25349325 PMC4256843

[jkad247-B37] De La Torre AR , Lin Y-C, Van de Peer Y, Ingvarsson PK. 2015. Genome-wide analysis reveals diverged patterns of codon bias, gene expression, and rates of sequence evolution in *Picea* gene families. Genome Biol Evol. 7(4):1002–1015. doi:10.1093/gbe/evv044. 25747252 PMC4419791

[jkad247-B38] De La Torre AR , Piot A, Liu B, Wilhite B, Weiss M, Porth I. 2020. Functional and morphological evolution in gymnosperms: a portrait of implicated gene families. Evol Appl. 13(1):210–227. doi:10.1111/eva.12839. 31892953 PMC6935586

[jkad247-B39] Depardieu C , Gérardi S, Nadeau S, Parent GJ, Mackay J, Lenz P, Lamothe M, Girardin MP, Bousquet J, Isabel N. 2021. Connecting tree-ring phenotypes, genetic associations and transcriptomics to decipher the genomic architecture of drought adaptation in a widespread conifer. Mol Ecol. 30(16):3898–3917. doi:10.1111/mec.15846. 33586257 PMC8451828

[jkad247-B40] de Vernal A , Hillaire-Marcel C. 2008. Natural variability of Greenland climate, vegetation, and ice volume during the past million years. Science. 320(5883):1622–1625. doi:10.1126/science.1153929. 18566284

[jkad247-B41] Di Franco A , Poujol R, Baurain D, Philippe H. 2019. Evaluating the usefulness of alignment filtering methods to reduce the impact of errors on evolutionary inferences. BMC Evol Biol. 19(1):21. doi:10.1186/s12862-019-1350-2. 30634908 PMC6330419

[jkad247-B42] Emms DM , Kelly S. 2019. Orthofinder: phylogenetic orthology inference for comparative genomics. Genom Biol. 20(1):238. doi:10.1186/s13059-019-1832-y. PMC6857279 31727128

[jkad247-B43] Ence D , Smith KE, Fan S, Gomide Neves L, Paul R, Wegrzyn J, Peter GF, Kirst M, Brawner J, Nelson CD, et al 2022. NLR Diversity and candidate fusiform rust resistance genes in loblolly pine. G3 (Bethesda). 12(2):jkab421. doi:10.1093/g3journal/jkab421. 34897455 PMC9210285

[jkad247-B44] Endo H , Yamaguchi M, Tamura T, Nakano Y, Nishikubo N, Yoneda A, Kato K, Kubo M, Kajita S, Katayama Y, et al 2015. Multiple classes of transcription factors regulate the expression of vascular-related NAC-domain7, a master switch of xylem vessel differentiation. Plant Cell Physiol. 56(2):242–254. doi:10.1093/pcp/pcu134. 25265867

[jkad247-B45] Falk T , Herndon N, Grau E, Buehler S, Richter P, Zaman S, Baker EM, Ramnath R, Ficklin S, Staton M, et al 2018. Growing and cultivating the forest genomics database, TreeGenes. Database (Oxford). 2018:1–11. doi:10.1093/database/bay084. PMC6146132 30239664

[jkad247-B46] Flynn JM , Hubley R, Goubert C, Rosen J, Clark AG, Feschotte C, Smit AF. 2020. Repeatmodeler2 for automated genomic discovery of transposable element families. Proc Natl Acad Sci U S A. 117(17):9451–9457. doi:10.1073/pnas.1921046117. 32300014 PMC7196820

[jkad247-B47] Gagalova KK , Warren RL, Coombe L, Wong J, Nip KM, Yuen MMS, Whitehill JGA, Celedon JM, Ritland C, Taylor GA, et al 2022. Spruce giga-genomes: structurally similar yet distinctive with differentially expanding gene families and rapidly evolving genes. Plant J. 111(5):1469–1485. doi:10.1111/tpj.15889. 35789009

[jkad247-B48] Gallemí M , Galstyan A, Pauliši S, Then C, Ferrández-Ayela A, Lorenzo-Orts L, Roig-Villanova I, Wang X, Micol JL, Ponce MR, et al 2016. DRACULA2 is a dynamic nucleoporin with a role in regulating the shade avoidance syndrome in *Arabidopsis*. Development. 143(9):1623–1631. doi:10.1242/dev.130211. 26989173

[jkad247-B49] Gauthier S , Bernier P, Kuuluvainen T, Shvidenko AZ, Schepaschenko DG. 2015. Boreal forest health and global change. Science. 349(6250):819–822. doi:10.1126/science.aaa9092. 26293953

[jkad247-B50] Geib SM , Hall B, Derego T, Bremer FT, Cannoles K, Sim SB. 2018. Genome annotation generator: a simple tool for generating and correcting WGS annotation tables for NCBI submission. GigaScience. 7(4):giy018. doi:10.1093/gigascience/giy018. 29635297 PMC5887294

[jkad247-B51] Gérardi S , Jaramillo-Correa JP, Beaulieu J, Bousquet J. 2010. From glacial refugia to modern populations: new assemblages of organelle genomes generated by differential cytoplasmic gene flow in transcontinental black spruce. Mol Ecol. 19(23):5265–5280. doi:10.1111/j.1365-294X.2010.04881.x. 21044193

[jkad247-B52] Gonzalez-Ibeas D , Martinez-Garcia PJ, Famula RA, Delfino-Mix A, Stevens KA, Loopstra CA, Langley CH, Neale DB, Wegrzyn JL. 2016. Assessing the gene content of the megagenome: sugar pine (*Pinus lambertiana*). G3 (Bethesda). 6(12):3787–3802. doi:10.1534/g3.116.032805. 27799338 PMC5144951

[jkad247-B53] Gurevich A , Saveliev V, Vyahhi N, Tesler G. 2013. QUAST: quality assessment tool for genome assemblies. Bioinformatics. 29(8):1072–1075. doi:10.1093/bioinformatics/btt086. 23422339 PMC3624806

[jkad247-B54] Hart AJ , Ginzburg S, Xu M, Fisher CR, Rahmatpour N, Mitton JB, Paul R, Wegrzyn JL. 2020. EnTAP: bringing faster and smarter functional annotation to non-model eukaryotic transcriptomes. Mol Ecol Resour. 20(2):591–604. doi:10.1111/1755-0998.13106. 31628884

[jkad247-B55] Hoffmann AA , Hercus MJ. 2000. Environmental stress as an evolutionary force. BioScience. 50(3):217–226. doi:10.1641/0006-3568(2000)050[0217:ESAAEF]2.3.CO;2.

[jkad247-B56] Hornoy B , Pavy N, Gérardi S, Beaulieu J, Bousquet J. 2015. Genetic adaptation to climate in white spruce involves small to moderate allele frequency shifts in functionally diverse genes. Genome Biol Evol. 7(12):3269–3285. doi:10.1093/gbe/evv218. 26560341 PMC4700950

[jkad247-B57] Huang K-C , Lin W-C, Cheng W-H. 2018. Salt hypersensitive mutant 9, a nucleolar APUM23 protein, is essential for salt sensitivity in association with the ABA signaling pathway in *Arabidopsis*. BMC Plant Biol. 18(1):40. doi:10.1186/s12870-018-1255-z. 29490615 PMC5831739

[jkad247-B58] Isabel N , Holliday JA, Aitken SN. 2020. Forest genomics: advancing climate adaptation, forest health, productivity, and conservation. Evol Appl. 13(1):3–10. doi:10.1111/eva.12902. 31892941 PMC6935596

[jkad247-B59] Jackman SD , Coombe L, Chu J, Warren RL, Vandervalk BP, Yeo S, Xue Z, Mohamadi H, Bohlmann J, Jones SJM, et al 2018. Tigmint: correcting assembly errors using linked reads from large molecules. BMC Bioinform. 19(1):393. doi:10.1186/s12859-018-2425-6. PMC6204047 30367597

[jkad247-B60] Jackman SD , Coombe L, Warren RL, Kirk H, Trinh E, MacLeod T, Pleasance S, Pandoh P, Zhao Y, Coope RJ, et al 2020. Complete mitochondrial genome of a gymnosperm, Sitka spruce (*Picea sitchensis*), indicates a complex physical structure. Genome Biol Evol. 12(7):1174–1179. doi:10.1093/gbe/evaa108. 32449750 PMC7486957

[jkad247-B61] Jackman SD , Vandervalk BP, Mohamadi H, Chu J, Yeo S, Hammond SA, Jahesh G, Khan H, Coombe L, Warren RL, et al 2017. ABySS 2.0: resource-efficient assembly of large genomes using a Bloom filter. Genome Res. 27(5):768–777. doi:10.1101/gr.214346.116. 28232478 PMC5411771

[jkad247-B62] Jackman SD , Warren RL, Gibb EA, Vandervalk BP, Mohamadi H, Chu J, Raymond A, Pleasance S, Coope R, Wildung MR, et al 2015. Organellar genomes of white spruce (*Picea glauca*): assembly and annotation. Genome Biol Evol. 8(1):29–41. doi:10.1093/gbe/evv244. 26645680 PMC4758241

[jkad247-B63] Jaramillo-Correa JP , Beaulieu J, Bousquet J. 2004. Variation in mitochondrial DNA reveals multiple distant glacial refugia in black spruce (*Picea mariana*), a transcontinental North American conifer. Mol Ecol. 13(9):2735–2747. doi:10.1111/j.1365-294X.2004.02258.x. 15315685

[jkad247-B64] Kaplan-Levy RN , Brewer PB, Quon T, Smyth DR. 2012. The trihelix family of transcription factors—light, stress and development. Trends Plant Sci. 17(3):163–171. doi:10.1016/j.tplants.2011.12.002. 22236699

[jkad247-B65] Katz LS , Griswold T, Morrison SS, Caravas JA, Zhang S, den Bakker HC, Deng X, Carleton HA. 2019. Mashtree: a rapid comparison of whole genome sequence files. J Open Source Softw. 4(44):1762. doi:10.21105/joss.01762. PMC9380445 35978566

[jkad247-B66] Kawasaki T , Yamada K, Yoshimura S, Yamaguchi K. 2017. Chitin receptor-mediated activation of MAP kinases and ROS production in rice and *Arabidopsis*. Plant Signal Behav. 12(9):e1361076. doi:10.1080/15592324.2017.1361076. 28805500 PMC5640189

[jkad247-B67] Kharenko OA , Boyd J, Nelson KM, Abrams SR, Loewen MC. 2011. Identification and characterization of interactions between abscisic acid and mitochondrial adenine nucleotide translocators. Biochem J. 437(1):117–123. doi:10.1042/BJ20101898. 21473740

[jkad247-B68] Kim D , Langmead B, Salzberg SL. 2015. HISAT: a fast spliced aligner with low memory requirements. Nat Methods. 12(4):357–360. doi:10.1038/nmeth.3317. 25751142 PMC4655817

[jkad247-B69] Kimura M . 1977. Preponderance of synonymous changes as evidence for the neutral theory of molecular evolution. Nature. 267(5608):275–276. doi:10.1038/267275a0. 865622

[jkad247-B70] Kobayashi K , Fujii S, Sasaki D, Baba S, Ohta H, Masuda T, Wada H. 2014. Transcriptional regulation of thylakoid galactolipid biosynthesis coordinated with chlorophyll biosynthesis during the development of chloroplasts in *Arabidopsis*. Front Plant Sci. 5:272. doi:10.3389/fpls.2014.00272. 24966866 PMC4052731

[jkad247-B71] Kriventseva EV , Kuznetsov D, Tegenfeldt F, Manni M, Dias R, et al 2019. OrthoDB v10: sampling the diversity of animal, plant, fungal, protist, bacterial and viral genomes for evolutionary and functional annotations of orthologs. Nucleic Acids Res. 47:D807–D811. 30395283 10.1093/nar/gky1053 PMC6323947

[jkad247-B72] Kryazhimskiy S , Plotkin JB. 2008. The population genetics of dN/dS. PLoS Genet. 4(12):e1000304. doi:10.1371/journal.pgen.1000304. 19081788 PMC2596312

[jkad247-B73] Kuromori T , Wada T, Kamiya A, Yuguchi M, Yokouchi T, Imura Y, Takabe H, Sakurai T, Akiyama K, Hirayama T, et al 2006. A trial of phenome analysis using 4000 Ds-insertional mutants in gene-coding regions of *Arabidopsis*. Plant J. 47(4):640–651. doi:10.1111/j.1365-313X.2006.02808.x. 16813574

[jkad247-B74] Laberge M-J , Payette S, Bousquet J. 2000. Life span and biomass allocation of stunted black spruce clones in the subartic environment. J Ecol. 88(4):584–593. doi:10.1046/j.1365-2745.2000.00471.x.

[jkad247-B75] Lannoo N , Van Damme EJM. 2014. Lectin domains at the frontiers of plant defense. Front Plant Sci. 5:397. doi:10.3389/fpls.2014.00397. 25165467 PMC4131498

[jkad247-B76] Laverdière J-P , Lenz P, Nadeau S, Depardieu C, Isabel N, Perron M, Beaulieu J, Bousquet J. 2022. Breeding for adaptation to climate change: genomic selection for drought response in a white spruce multi-site polycross test. Evol Appl. 15(3):383–402. doi:10.1111/eva.13348. 35386396 PMC8965362

[jkad247-B77] Lenz PRN , Beaulieu J, Mansfield SD, Clément S, Desponts M, Bousquet J. 2017. Factors affecting the accuracy of genomic selection for growth and wood quality traits in an advanced-breeding population of black spruce (*Picea mariana*). BMC Genomics. 18(1):335. doi:10.1186/s12864-017-3715-5. 28454519 PMC5410046

[jkad247-B78] Li H . 2014. lh3/trimadap [Internet]. [cited 2022 Aug 10]. Available from https://github.com/lh3/trimadap

[jkad247-B79] Li L , Foster CM, Gan Q, Nettleton D, James MG, Myers AM, Wurtele ES. 2009. Identification of the novel protein QQS as a component of the starch metabolic network in *Arabidopsis* leaves. Plant J. 58(3):485–498. doi:10.1111/j.1365-313X.2009.03793.x. 19154206

[jkad247-B80] Li C-H , Wang G, Zhao J-L, Zhang L-Q, Ai L-F, Han Y-F, Sun D-Y, Zhang S-W, Sun Y. 2014. The receptor-like kinase SIT1 mediates salt sensitivity by activating MAPK3/6 and regulating ethylene homeostasis in rice. Plant Cell. 26(6):2538–2553. doi:10.1105/tpc.114.125187. 24907341 PMC4114950

[jkad247-B81] Lin D , Coombe L, Jackman SD, Gagalova KK, Warren RL, Hammond SA, Kirk H, Pandoh P, Zhao Y, Moore RA, et al 2019a. Complete chloroplast genome sequence of a white spruce (*Picea glauca*, genotype WS77111) from eastern Canada. Microbiol Resour Announc. 8(23):e00381–e00319. doi:10.1128/MRA.00381-19. 31171622 PMC6554609

[jkad247-B82] Lin D , Coombe L, Jackman SD, Gagalova KK, Warren RL, Hammond SA, McDonald H, Kirk H, Pandoh P, Zhao Y, et al 2019b. Complete chloroplast genome sequence of an Engelmann spruce (*Picea engelmannii*, genotype Se404–851) from western Canada. Microbiol Resour Announc. 8(24):e00382–e00319. doi:10.1128/MRA.00382-19. 31196920 PMC6588038

[jkad247-B83] Lo T , Coombe L, Lin D, Warren RL, Kirk H, Pandoh P, Zhao Y, Moore RA, Mungall AJ, Ritland C, et al 2020. Complete chloroplast genome sequence of a black spruce (*Picea mariana*) from eastern Canada. Microbiol Resour Announc. 9(39):e00877–e00820. doi:10.1128/MRA.00877-20. 32972944 PMC7516155

[jkad247-B84] Lo T , Gagalova KK, Coombe L, Warren RL, Birol I. 2023. Picea mariana isolate 40–10-1 genome annotation. Zenodo. doi:10.5281/zenodo.7830121.

[jkad247-B85] MacLachlan IR , McDonald TK, Lind BM, Rieseberg LH, Yeaman S, Aitken SN. 2021. Genome-wide shifts in climate-related variation underpin responses to selective breeding in a widespread conifer. Proc Natl Acad Sci U S A. 118(10):e2016900118. doi:10.1073/pnas.2016900118. PMC7958292 33649218

[jkad247-B86] Mann IK , Wegrzyn JL, Rajora OP. 2013. Generation, functional annotation and comparative analysis of black spruce (*Picea mariana*) ESTs: an important conifer genomic resource. BMC Genom. 14(1):702. doi:10.1186/1471-2164-14-702. PMC4007752 24119028

[jkad247-B87] Marr A , Coombe L, Warren RL, Birol I. 2023. Picea mariana isolate 40-10-1 mitochondrial genome assembly. Zenodo. doi:10.5281/zenodo.7828188.

[jkad247-B88] Mathur S , Vyas S, Kapoor S, Tyagi AK. 2011. The mediator complex in plants: structure, phylogeny, and expression profiling of representative genes in a dicot (*Arabidopsis*) and a monocot (rice) during reproduction and abiotic stress. Plant Physiol. 157(4):1609–1627. doi:10.1104/pp.111.188300. 22021418 PMC3327187

[jkad247-B89] Michaels SD , Bezerra IC, Amasino RM. 2004. FRIGIDA-related genes are required for the winter-annual habit in *Arabidopsis*. Proc Natl Acad Sci U S A. 101(9):3281–3285. doi:10.1073/pnas.0306778101. 14973192 PMC365781

[jkad247-B90] Mølhøj M , Jørgensen B, Ulvskov P, Borkhardt B. 2001. Two *Arabidopsis thaliana* genes, KOR2 and KOR3, which encode membrane-anchored endo-1,4-beta-D-glucanases, are differentially expressed in developing leaf trichomes and their support cells. Plant Mol Biol. 46(3):263–275. doi:10.1023/a:1010688726755. 11488474

[jkad247-B91] Mullin TJ , Andersson B, Bastien J-C, Beaulieu J, Burdon RD, Dvorak WS, King JN, Kondo T, Krakowski J, Lee SJ, et al 2011. Economic importance, breeding objectives and achievements. In: Plomion C, Bousquet J, Kole C, editors. Genetics, Genomics, and Breeding of Conifers. New York (NY): CRC Press. p. 456 p.

[jkad247-B92] Namroud M-C , Beaulieu J, Juge N, Laroche J, Bousquet J. 2008. Scanning the genome for gene single nucleotide polymorphisms involved in adaptive population differentiation in white spruce. Mol Ecol. 17(16):3599–3613. doi:10.1111/j.1365-294X.2008.03840.x. 18662225 PMC2613251

[jkad247-B93] Neale DB , Kremer A. 2011. Forest tree genomics: growing resources and applications. Nat Rev Genet. 12(2):111–122. doi:10.1038/nrg2931. 21245829

[jkad247-B94] Neale DB , Wegrzyn JL, Stevens KA, Zimin AV, Puiu D, Crepeau MW, Cardeno C, Koriabine M, Holtz-Morris AE, Liechty JD, et al 2014. Decoding the massive genome of loblolly pine using haploid DNA and novel assembly strategies. Genome Biol. 15(3):R59. doi:10.1186/gb-2014-15-3-r59. 24647006 PMC4053751

[jkad247-B95] Neale DB , Zimin AV, Zaman S, Scott AD, Shrestha B, Workman RE, Puiu D, Allen BJ, Moore ZJ, Sekhwal MK, et al 2022. Assembled and annotated 26.5 Gbp coast redwood genome: a resource for estimating evolutionary adaptive potential and investigating hexaploid origin. G3 (Bethesda). 12(1):jkab380. doi:10.1093/g3journal/jkab380. 35100403 PMC8728005

[jkad247-B96] Nicklen EF , Roland CA, Ruess RW, Scharnweber T, Wilmking M. 2021. Divergent responses to permafrost and precipitation reveal mechanisms for the spatial variation of two sympatric spruce. Ecosphere. 12(7):e03622. doi:10.1002/ecs2.3622.

[jkad247-B97] Niu S , Li J, Bo W, Yang W, Zuccolo A, Giacomello S, Chen X, Han F, Yang J, Song Y, et al 2022. The Chinese pine genome and methylome unveil key features of conifer evolution. Cell. 185(1):204–217.e14. doi:10.1016/j.cell.2021.12.006. 34965378

[jkad247-B98] Nystedt B , Street NR, Wetterbom A, Zuccolo A, Lin Y-C, Scofield DG, Vezzi F, Delhomme N, Giacomello S, Alexeyenko A, et al 2013. The Norway spruce genome sequence and conifer genome evolution. Nature. 497(7451):579–584. doi:10.1038/nature12211. 23698360

[jkad247-B99] Okushima Y , Fukaki H, Onoda M, Theologis A, Tasaka M. 2007. ARF7 And ARF19 regulate lateral root formation via direct activation of LBD/ASL genes in *Arabidopsis*. Plant Cell. 19(1):118–130. doi:10.1105/tpc.106.047761. 17259263 PMC1820965

[jkad247-B100] Okushima Y , Overvoorde PJ, Arima K, Alonso JM, Chan A, Chang C, Ecker JR, Hughes B, Lui A, Nguyen D, et al 2005. Functional genomic analysis of the auxin response factor gene family members in *Arabidopsis thaliana*: unique and overlapping functions of ARF7 and ARF19. Plant Cell. 17(2):444–463. doi:10.1105/tpc.104.028316. 15659631 PMC548818

[jkad247-B101] O’Leary NA , Wright MW, Brister JR, Ciufo S, Haddad D, McVeigh R, Rajput B, Robbertse B, Smith-White B, Ako-Adjei D, et al 2016. Reference sequence (RefSeq) database at NCBI: current status, taxonomic expansion, and functional annotation. Nucleic Acids Res. 44(D1):D733–D745. doi:10.1093/nar/gkv1189. 26553804 PMC4702849

[jkad247-B102] Otsuka K , Mamiya A, Konishi M, Nozaki M, Kinoshita A, Tamaki H, Arita M, Saito M, Yamamoto K, Hachiya T, et al 2021. Temperature-dependent fasciation mutants provide a link between mitochondrial RNA processing and lateral root morphogenesis. Elife. 10:e61611. doi:10.7554/eLife.61611. 33443014 PMC7846275

[jkad247-B103] Ou S , Jiang N. 2018. LTR_Retriever: a highly accurate and sensitive program for identification of long terminal repeat retrotransposons. Plant Physiol. 176(2):1410–1422. doi:10.1104/pp.17.01310. 29233850 PMC5813529

[jkad247-B104] Patro R , Duggal G, Love MI, Irizarry RA, Kingsford C. 2017. Salmon: fast and bias-aware quantification of transcript expression using dual-phase inference. Nat Methods. 14(4):417–419. doi:10.1038/nmeth.4197. 28263959 PMC5600148

[jkad247-B105] Paulino D , Warren RL, Vandervalk BP, Raymond A, Jackman SD, Birol I. 2015. Sealer: a scalable gap-closing application for finishing draft genomes. BMC Bioinform. 16(1):230. doi:10.1186/s12859-015-0663-4. PMC4515008 26209068

[jkad247-B106] Pavy N , Gagnon F, Deschênes A, Boyle B, Beaulieu J, Bousquet J. 2016. Development of highly reliable in silico SNP resource and genotyping assay from exome capture and sequencing: an example from black spruce (*Picea mariana*). Mol Ecol Resour. 16(2):588–598. doi:10.1111/1755-0998.12468. 26391535

[jkad247-B107] Pavy N , Pelgas B, Beauseigle S, Blais S, Gagnon F, Gosselin I, Lamothe M, Isabel N, Bousquet J. 2008. Enhancing genetic mapping of complex genomes through the design of highly-multiplexed SNP arrays: application to the large and unsequenced genomes of white spruce and black spruce. BMC Genom. 9(1):21. doi:10.1186/1471-2164-9-21. PMC2246113 18205909

[jkad247-B108] Pelgas B , Bousquet J, Meirmans PG, Ritland K, Isabel N. 2011. QTL mapping in white spruce: gene maps and genomic regions underlying adaptive traits across pedigrees, years and environments. BMC Genom. 12(1):145. doi:10.1186/1471-2164-12-145. PMC3068112 21392393

[jkad247-B109] Plomion C , Bastien C, Bogeat-Triboulot M-B, Bouffier L, Déjardin A, Duplessis S, Fady B, Heuertz M, Le Gac A-L, Le Provost G, et al 2016. Forest tree genomics: 10 achievements from the past 10 years and future prospects. Ann For Sci. 73(1):77–103. doi:10.1007/s13595-015-0488-3.

[jkad247-B110] Proost S , Van Bel M, Sterck L, Billiau K, Van Parys T, Van de Peer Y, Vandepoele K. 2009. PLAZA: a comparative genomics resource to study gene and genome evolution in plants. Plant Cell. 21(12):3718–3731. doi:10.1105/tpc.109.071506. 20040540 PMC2814516

[jkad247-B111] Prunier J , Gérardi S, Laroche J, Beaulieu J, Bousquet J. 2012. Parallel and lineage-specific molecular adaptation to climate in boreal black spruce. Mol Ecol. 21(17):4270–4286. doi:10.1111/j.1365-294X.2012.05691.x. 22805595

[jkad247-B112] Prunier J , Laroche J, Beaulieu J, Bousquet J. 2011. Scanning the genome for gene SNPs related to climate adaptation and estimating selection at the molecular level in boreal black spruce. Mol Ecol. 20(8):1702–1716. doi:10.1111/j.1365-294X.2011.05045.x. 21375634

[jkad247-B113] Prunier J , Pelgas B, Gagnon F, Desponts M, Isabel N, Beaulieu J, Bousquet J. 2013. The genomic architecture and association genetics of adaptive characters using a candidate SNP approach in boreal black spruce. BMC Genom. 14(1):368. doi:10.1186/1471-2164-14-368. PMC3674900 23724860

[jkad247-B114] Ran J-H , Shen T-T, Liu W-J, Wang P-P, Wang X-Q. 2015. Mitochondrial introgression and complex biogeographic history of the genus *Picea*. Mol Phylogenet Evol. 93:63–76. doi:10.1016/j.ympev.2015.07.020. 26232548

[jkad247-B115] Ran J-H , Wei X-X, Wang X-Q. 2006. Molecular phylogeny and biogeography of *Picea* (Pinaceae): implications for phylogeographical studies using cytoplasmic haplotypes. Mol Phylogenet Evol. 41(2):405–419. doi:10.1016/j.ympev.2006.05.039. 16839785

[jkad247-B116] Ranwez V , Douzery EJP, Cambon C, Chantret N, Delsuc F. 2018. MACSE V2: toolkit for the alignment of coding sequences accounting for frameshifts and stop codons. Mol Biol Evol. 35(10):2582–2584. doi:10.1093/molbev/msy159. 30165589 PMC6188553

[jkad247-B117] Rao V , Virupapuram V. 2021. Arabidopsis F-box protein At1g08710 interacts with transcriptional protein ADA2b and imparts drought stress tolerance by negatively regulating seedling growth. Biochem Biophys Res Commun. 536:45–51. doi:10.1016/j.bbrc.2020.12.054. 33360542

[jkad247-B118] Riechmann JL , Heard J, Martin G, Reuber L, Jiang C, Keddie J, Adam L, Pineda O, Ratcliffe OJ, Samaha RR, et al 2000. *Arabidopsis* transcription factors: genome-wide comparative analysis among eukaryotes. Science. 290(5499):2105–2110. doi:10.1126/science.290.5499.2105. 11118137

[jkad247-B119] Savolainen O , Lascoux M, Merilä J. 2013. Ecological genomics of local adaptation. Nat Rev Genet. 14(11):807–820. doi:10.1038/nrg3522. 24136507

[jkad247-B120] Schneider A , Souvorov A, Sabath N, Landan G, Gonnet GH, Graur D. 2009. Estimates of positive Darwinian selection are inflated by errors in sequencing, annotation, and alignment. Genome Biol Evol. 1:114–118. doi:10.1093/gbe/evp012. 20333182 PMC2817407

[jkad247-B121] Scott AD , Zimin AV, Puiu D, Workman R, Britton M, Zaman S, Caballero M, Read AC, Bogdanove AJ, Burns E, et al 2020. A reference genome sequence for giant sequoia. G3 (Bethesda). 10(11):3907–3919. doi:10.1534/g3.120.401612. 32948606 PMC7642918

[jkad247-B122] Shao C-C , Shen T-T, Jin W-T, Mao H-J, Ran J-H, Wang X-Q. 2019. Phylotranscriptomics resolves interspecific relationships and indicates multiple historical out-of-North America dispersals through the Bering Land Bridge for the genus *Picea* (Pinaceae). Mol Phylogenet Evol. 141:106610. doi:10.1016/j.ympev.2019.106610. 31499190

[jkad247-B123] Shen G , Kuppu S, Venkataramani S, Wang J, Yan J, Qiu X, Zhang H. 2010. Ankyrin repeat-containing protein 2A is an essential molecular chaperone for peroxisomal membrane-bound ascorbate peroxidase3 in *Arabidopsis*. Plant Cell. 22(3):811–831. doi:10.1105/tpc.109.065979. 20215589 PMC2861468

[jkad247-B124] Simão FA , Waterhouse RM, Ioannidis P, Kriventseva EV, Zdobnov EM. 2015. BUSCO: assessing genome assembly and annotation completeness with single-copy orthologs. Bioinformatics. 31(19):3210–3212. doi:10.1093/bioinformatics/btv351. 26059717

[jkad247-B125] Singh P , Zimmerli L. 2013. Lectin receptor kinases in plant innate immunity. Front Plant Sci. 4:124. doi:10.3389/fpls.2013.00124. 23675375 PMC3646242

[jkad247-B126] Stanke M , Keller O, Gunduz I, Hayes A, Waack S, Morgenstern B. 2006. AUGUSTUS: ab initio prediction of alternative transcripts. Nucleic Acids Res. 34(Web Server):W435–W439. doi:10.1093/nar/gkl200. 16845043 PMC1538822

[jkad247-B127] Steinegger M , Söding J. 2017. MMseqs2 enables sensitive protein sequence searching for the analysis of massive data sets. Nat Biotechnol. 35(11):1026–1028. doi:10.1038/nbt.3988. 29035372

[jkad247-B128] Stival Sena J , Giguère I, Boyle B, Rigault P, Birol I, Zuccolo A, Ritland K, Ritland C, Bohlmann J, Jones S, et al 2014. Evolution of gene structure in the conifer *Picea glauca*: a comparative analysis of the impact of intron size. BMC Plant Biol. 14(1):95. doi:10.1186/1471-2229-14-95. 24734980 PMC4108047

[jkad247-B129] Stival Sena J , Giguère I, Rigault P, Bousquet J, Mackay J. 2018. Expansion of the dehydrin gene family in the Pinaceae is associated with considerable structural diversity and drought-responsive expression. Tree Physiol. 38(3):442–456. doi:10.1093/treephys/tpx125. 29040752

[jkad247-B130] Sullivan AR , Eldfjell Y, Schiffthaler B, Delhomme N, Asp T, Hebelstrup KH, Keech O, Öberg L, Møller IM, Arvestad L, et al 2020. The mitogenome of Norway spruce and a reappraisal of mitochondrial recombination in plants. Genome Biol Evol. 12(1):3586–3598. doi:10.1093/gbe/evz263. 31774499 PMC6944214

[jkad247-B131] Sullivan AR , Schiffthaler B, Thompson SL, Street NR, Wang X-R. 2017. Interspecific plastome recombination reflects ancient reticulate evolution in Picea (Pinaceae). Mol Biol Evol. 34(7):1689–1701. doi:10.1093/molbev/msx111. 28383641 PMC5455968

[jkad247-B132] Suzek BE , Wang Y, Huang H, McGarvey PB, Wu CH; UniProt Consortium. 2015. Uniref clusters: a comprehensive and scalable alternative for improving sequence similarity searches. Bioinformatics. 31(6):926–932. doi:10.1093/bioinformatics/btu739. 25398609 PMC4375400

[jkad247-B133] Tabas-Madrid D , Méndez-Vigo B, Arteaga N, Marcer A, Pascual-Montano A, Weigel D, Xavier Picó F, Alonso-Blanco C. 2018. Genome-wide signatures of flowering adaptation to climate temperature: regional analyses in a highly diverse native range of *Arabidopsis thaliana*. Plant Cell Environ. 41(8):1806–1820. doi:10.1111/pce.13189. 29520809

[jkad247-B134] Tamura K , Stecher G, Kumar S. 2021. MEGA11: molecular evolutionary genetics analysis version 11. Mol Biol Evol. 38(7):3022–3027. doi:10.1093/molbev/msab120. 33892491 PMC8233496

[jkad247-B135] Tenhaken R , Doerks T, Bork P. 2005. DCD—a novel plant specific domain in proteins involved in development and programmed cell death. BMC Bioinform. 6(1):169. doi:10.1186/1471-2105-6-169. PMC1182354 16008837

[jkad247-B136] The UniProt Consortium . 2019. Uniprot: a worldwide hub of protein knowledge. Nucleic Acids Res. 47(D1):D506–D515. doi:10.1093/nar/gky1049. 30395287 PMC6323992

[jkad247-B137] Thomson AM , Riddell CL, Parker WH. 2009. Boreal forest provenance tests used to predict optimal growth and response to climate change: 2. Black spruce. Can J For Res. 39(1):143–153. doi:10.1139/X08-167.

[jkad247-B138] Trujillo-Moya C , George J-P, Fluch S, Geburek T, Grabner M, Karanitsch-Ackerl S, Konrad H, Mayer K, Sehr EM, Wischnitzki E, et al 2018. Drought sensitivity of Norway spruce at the species’ warmest fringe: quantitative and molecular analysis reveals high genetic variation among and within provenances. G3 (Bethesda). 8(4):1225–1245. doi:10.1534/g3.117.300524. 29440346 PMC5873913

[jkad247-B139] Tunc-Ozdemir M , Rato C, Brown E, Rogers S, Mooneyham A, Frietsch S, Myers CT, Poulsen LR, Malhó R, Harper JF. 2013. Cyclic nucleotide gated channels 7 and 8 are essential for male reproductive fertility. PLoS One. 8(2):e55277. doi:10.1371/journal.pone.0055277. 23424627 PMC3570425

[jkad247-B140] Vandervalk BP , Yang C, Xue Z, Raghavan K, Chu J, Mohamadi H, Jackman SD, Chiu R, Warren RL, Birol I. 2015. Konnector v2.0: pseudo-long reads from paired-end sequencing data. BMC Med Genom. 8(S3):S1. doi:10.1186/1755-8794-8-S3-S1. PMC4582294 26399504

[jkad247-B141] Van Ghelder C , Parent GJ, Rigault P, Prunier J, Giguère I, Caron S, Stival Sena J, Deslauriers A, Bousquet J, Esmenjaud D, et al 2019. The large repertoire of conifer NLR resistance genes includes drought responsive and highly diversified RNLs. Sci Rep. 9(1):11614. doi:10.1038/s41598-019-47950-7. 31406137 PMC6691002

[jkad247-B142] Västermark Å , Almén MS, Simmen MW, Fredriksson R, Schiöth HB. 2011. Functional specialization in nucleotide sugar transporters occurred through differentiation of the gene cluster EamA (DUF6) before the radiation of *Viridiplantae*. BMC Evol Biol. 11(1):123. doi:10.1186/1471-2148-11-123. 21569384 PMC3111387

[jkad247-B143] Venkatraman M , Fleischer RC, Tsuchiya MTN. 2021. Comparative analysis of annotation pipelines using the first Japanese white-eye (*Zosterops japonicus*) genome. Genome Biol Evol. 13(5):evab063. doi:10.1093/gbe/evab063. 33760049 PMC8120012

[jkad247-B144] Vidali L , van Gisbergen PAC, Guérin C, Franco P, Li M, Burkart GM, Augustine RC, Blanchoin L, Bezanilla M. 2009. Rapid formin-mediated actin-filament elongation is essential for polarized plant cell growth. Proc Natl Acad Sci U S A. 106(32):13341–13346. doi:10.1073/pnas.0901170106. 19633191 PMC2726404

[jkad247-B145] Vuruputoor VS , Monyak D, Fetter KC, Webster C, Bhattarai A, Shrestha B, Zaman S, Bennett J, McEvoy SL, Caballero M, et al 2023. Welcome to the big leaves: best practices for improving genome annotation in non-model plant genomes. Appl Plant Sci. 11(4):e11533. doi:10.1002/aps3.11533. 37601314 PMC10439824

[jkad247-B146] Wan J , Patel A, Mathieu M, Kim S-Y, Xu D, Stacey G. 2008. A lectin receptor-like kinase is required for pollen development in *Arabidopsis*. Plant Mol Biol. 67(5):469–482. doi:10.1007/s11103-008-9332-6. 18392777

[jkad247-B147] Wang K , Guo Q, Froehlich JE, Hersh HL, Zienkiewicz A, Howe GA, Benning C. 2018. Two abscisic acid-responsive plastid lipase genes involved in jasmonic acid biosynthesis in *Arabidopsis thaliana*. Plant Cell. 30(5):1006–1022. doi:10.1105/tpc.18.00250. 29666162 PMC6002186

[jkad247-B148] Wang Y , Liu K, Liao H, Zhuang C, Ma H, Yan X. 2008. The plant WNK gene family and regulation of flowering time in *Arabidopsis*. Plant Biol (Stuttg). 10(5):548–562. doi:10.1111/j.1438-8677.2008.00072.x. 18761494

[jkad247-B149] Warren RL , Keeling CI, Yuen MMS, Raymond A, Taylor GA, Vandervalk BP, Mohamadi H, Paulino D, Chiu R, Jackman SD, et al 2015. Improved white spruce (*Picea glauca*) genome assemblies and annotation of large gene families of conifer terpenoid and phenolic defense metabolism. Plant J. 83(2):189–212. doi:10.1111/tpj.12886. 26017574

[jkad247-B150] Warren RL , Yang C, Vandervalk BP, Behsaz B, Lagman A, Jones SJM, Birol I. 2015. LINKS: scalable, alignment-free scaffolding of draft genomes with long reads. GigaScience. 4(1):35. doi:10.1186/s13742-015-0076-3. 26244089 PMC4524009

[jkad247-B151] Whelan S , Irisarri I, Burki F. 2018. PREQUAL: detecting non-homologous characters in sets of unaligned homologous sequences. Bioinformatics. 34(22):3929–3930. doi:10.1093/bioinformatics/bty448. 29868763

[jkad247-B152] Wu Y , Xun Q, Guo Y, Zhang J, Cheng K, Shi T, He K, Hou S, Gou X, Li J. 2016. Genome-wide expression pattern analyses of the *Arabidopsis* leucine-rich repeat receptor-like kinases. Mol Plant. 9(2):289–300. doi:10.1016/j.molp.2015.12.011. 26712505

[jkad247-B153] Xue B , Charest PJ, Devantier Y, Rutledge RG. 2003. Characterization of a MYBR2R3 gene from black spruce (*Picea mariana*) that shares functional conservation with maize C1. Mol Gen Genomics. 270(1):78–86. doi:10.1007/s00438-003-0898-z. 12920576

[jkad247-B154] Yandell M , Ence D. 2012. A beginner's guide to eukaryotic genome annotation. Nat Rev Genet. 13(5):329–342. doi:10.1038/nrg3174. 22510764

[jkad247-B155] Yang Z . 2007. PAML 4: phylogenetic analysis by maximum likelihood. Mol Biol Evol. 24(8):1586–1591. doi:10.1093/molbev/msm088. 17483113

[jkad247-B156] Yang Z , dos Reis M. 2011. Statistical properties of the branch-site test of positive selection. Mol Biol Evol. 28(3):1217–1228. doi:10.1093/molbev/msq303. 21087944

[jkad247-B157] Yeaman S , Hodgins KA, Lotterhos KE, Suren H, Nadeau S, Degner JC, Nurkowski KA, Smets P, Wang T, Gray LK, et al 2016. Convergent local adaptation to climate in distantly related conifers. Science. 353(6306):1431–1433. doi:10.1126/science.aaf7812. 27708038

[jkad247-B158] Yeo S , Coombe L, Warren RL, Chu J, Birol I. 2018. ARCS: scaffolding genome drafts with linked reads. Bioinformatics. 34(5):725–731. doi:10.1093/bioinformatics/btx675. 29069293 PMC6030987

[jkad247-B159] Yu D , Chen C, Chen Z. 2001. Evidence for an important role of WRKY DNA binding proteins in the regulation of NPR1 gene expression. Plant Cell. 13(7):1527–1540. doi:10.1105/tpc.010115. 11449049 PMC139550

[jkad247-B160] Zhang J . 2005. Evaluation of an improved branch-site likelihood method for detecting positive selection at the molecular level. Mol Biol Evol. 22(12):2472–2479. doi:10.1093/molbev/msi237. 16107592

[jkad247-B161] Zhao J-L , Zhang L-Q, Liu N, Xu S-L, Yue Z-L, Zhang L-L, Deng Z-P, Burlingame AL, Sun D-Y, Wang Z-Y, et al 2019. Mutual regulation of receptor-like kinase SIT1 and b’κ-PP2A shapes the early response of rice to salt stress. Plant Cell. 31(9):2131–2151. doi:10.1105/tpc.18.00706. 31221736 PMC6751134

[jkad247-B162] Zheng B , He H, Zheng Y, Wu W, McCormick S. 2014. An ARID domain-containing protein within nuclear bodies is required for sperm cell formation in *Arabidopsis thaliana*. PLoS Genet. 10(7):e1004421. doi:10.1371/journal.pgen.1004421. 25057814 PMC4109846

[jkad247-B163] Zimin AV , Stevens KA, Crepeau MW, Puiu D, Wegrzyn JL, Yorke JA, Langley CH, Neale DB, Salzberg SL. 2017. An improved assembly of the loblolly pine mega-genome using long-read single-molecule sequencing. Gigascience. 6(1):1–4. doi:10.1093/gigascience/giw016. PMC5437942 28369353

