# Supplementary material for: Assembly and annotation of the black spruce genome provide insights on spruce phylogeny and evolution of stress response
Source: G3 (Bethesda). 2023 Oct 24;14(1):jkad247. doi: 10.1093/g3journal/jkad247 (PMC10755193; doi:10.1093/g3journal/jkad247)
Supplement: jkad247_Supplementary_Data [file jkad247_supplementary_data.zip › File_S1_G3-2023-404575.docx]

**Supplementary material for: Assembly and annotation of the black spruce genome provide insights on spruce phylogeny and evolution of stress response**

# **Authors:** Theodora Lo^1^, Lauren Coombe^1^, Kristina K. Gagalova^1^, Alex Marr^1^, René L. Warren^1^, Heather Kirk^1^, Pawan Pandoh^1^, Yongjun Zhao^1^, Richard A. Moore^1^, Andrew J. Mungall^1^, Carol Ritland^2,4^, Nathalie Pavy^3^, Steven J. M. Jones^1^, Joerg Bohlmann^2,4,5^, Jean Bousquet^3^, Inanç Birol^1,∗^ and Ashley Thomson^6,∗^

**Affiliations:**

^1^Canada’s Michael Smith Genome Sciences Centre, BC Cancer, Vancouver, BC V5Z 4S6, Canada

^2^Department of Forest and Conservation Sciences, University of British Columbia, Vancouver, BC V6T 1Z4, Canada

^3^Canada Research Chair in Forest Genomics, Laval University, Quebec City, QC G1V 0A6, Canada

^4^Michael Smith Laboratories, University of British Columbia, Vancouver, BC V6T 1Z4, Canada

^5^Department of Botany, University of British Columbia, Vancouver, BC V6T 1Z4, Canada

^6^Faculty of Natural Resources Management, Lakehead University, Thunder Bay, ON P7B 5E1, Canada

^∗^Address correspondence to Inanç Birol: [ibirol@bcgsc.ca](mailto:ibirol@bcgsc.ca) or to Ashley Thomson: [athomson@lakeheadu.ca](mailto:athomson@lakeheadu.ca)

Table of Contents

[Table S1. Sequencing data used for the assembly of *Picea mariana* nuclear genome 3](#_Toc146011551)

[Table S2. Summary of protein evidence provided to BRAKER 4](#_Toc146011552)

[Table S3. Summary of RNA evidence and resulting alignments provided to BRAKER 5](#_Toc146011553)

[Table S4. Number of protein-coding sequences and transcripts after each stage in the *Picea mariana* annotation pipeline 6](#_Toc146011554)

[Table S5. NCBI accessions of nuclear, chloroplast, and mitochondrial genome assemblies used in comparative genomics analysis 7](#_Toc146011555)

[Table S6. Assembly and annotations statistics for all spruce and pine nuclear genomes used in phylogenetic and comparative genomics analysis 8](#_Toc146011556)

[Table S7. Summary of OrthoFinder results 9](#_Toc146011557)

[Figure S1. Best matching PFAM domains (n > 5) in *Picea mariana* specific protein-coding sequences 10](#_Toc146011558)

# **Table S1.** Sequencing data used for the assembly of *Picea mariana* nuclear genome

.

| Platform | Read length (bp) | Estimated fragment size (bp) | Number of libraries | Number of sequencing lanes | Fold coverage | SRA accessions |
| --- | --- | --- | --- | --- | --- | --- |
| 10x Genomics Chromium | 2x150 | 400 | 1 | 5 | 31.15x | SRX7890468 |
| Illumina HiSeqX | 2x250 | 400 | 2 | 2 | 6.30x | SRX9351292, SRX9351293 |
| Illumina HiSeqX | 2x250 | 800 | 2 | 2 | 8.50x | SRX9351294, SRX9351295 |

# **Table S2.** Summary of protein evidence provided to BRAKER

obtained from the OrthoDB v10 Viridiplantae database, UniProtKB/Swissprot plant entries, Picea glauca manual annotations of terpene synthase and cytochrome P450 genes, and common proteins found in at least three of four North American spruces included in this analysis (interior spruce, Picea engelmannii. Picea glauca and Picea sitchensis), as identified by reciprocal-best hit (RBH) analysis. Specifically, reciprocal BLAST searches were performed between the North American spruce with the most annotations, Picea engelmannii, and each of the other three North American spruces. Proteins found in at least two of the three reciprocal BLAST search results were considered common amongst the North American spruces and included as evidence.

| Database | Number of proteins |
| --- | --- |
| OrthoDB v10 *Viridiplantae* | 3,510,742 |
| UniProtKB/SwissProt plant entries | 40,925 |
| *P. glauca* manual annotations | 298 |
| Common proteins in Canadian spruce annotations | 29,204 |
| Total number of unique proteins | **3,463,432** |

# **Table S3.** Summary of RNA evidence and resulting alignments provided to BRAKER

as annotation evidence. After obtaining the data from SRA, quality and contaminant filtering was performed prior to aligning the reads to the genome.

|  | SRR9595774 | SRR9595777 |
| --- | --- | --- |
| Total number of reads | 51,739,556 | 58,954,918 |
| Number of reads after fastp & contaminant-filtering | 43,337,856 | 53,418,516 |
| Number of aligned reads | 38,663,631 | 47,320,896 |
| Number of multi-mapped reads | 5,166,472 | 5,705,162 |

# **Table S4.** Number of protein-coding sequences and transcripts after each stage in the *Picea mariana* annotation pipeline

.

| Stage in annotation pipeline | Number of protein-coding sequences | Number of transcripts |
| --- | --- | --- |
| BRAKER | 146,065 | 150,036 |
| Remove incomplete (no start and stop codon) and/or intron lengths < 10 bp | 121,974 | 125,377 |
| EnTAP | 67,373 | 69,782 |
| Remove *gag* and *pol* Pfam domains | 66,332 | 68,738 |
| Identified as fragmented | 883 | 1,070 |

# **Table S5.** NCBI accessions of nuclear, chloroplast, and mitochondrial genome assemblies used in comparative genomics analysis

.

| Species | Nuclear genome | Chloroplast genome | Mitochondrial genome | References |
| --- | --- | --- | --- | --- |
| *Picea mariana* | JASDQU010000000 | MT261462 | [10.5281/zenodo.7828188](https://doi.org/10.5281/zenodo.7828187) | This study,  Lo *et al.* 2020 |
| Interior spruce | ALWZ000000000 | NC_028594 | LKAM01000001.1-LKAM01000036.1 | Gagalova *et al.* 2022,  Jackman *et al.* 2015 |
| *Picea abies* | GCA_900067695 | NC_021456 | MN642623-MN642626 | Nystedt *et al.* 2013 |
| *Picea engelmannii* | WSFP000000000 | NC_041067 | [10.5281/zenodo.7828263](https://doi.org/10.5281/zenodo.7828262) | Gagalova *et al.* 2022,  Lin *et al.* 2019b |
| *Picea glauca* | JZKD000000000 | MK174379 | [10.5281/zenodo.7828293](https://doi.org/10.5281/zenodo.7828292) | Gagalova *et al.* 2022,  Lin *et al.* 2019a |
| *Picea sitchensis* | SNQJ000000000 | KU215903 | MK697696-MK607708 | Gagalova *et al.* 2022,  Coombe *et al.* 2016,  Jackman *et al.* 2020 |
| *Pinus lambertiana* | GCA_001447015 | NC_011156 | <https://treegenesdb.org/FTP/Genomes/Pila/mito/plambertiana.mito.scafSeq.gz> | Gonzalez-Ibeas *et al.* 2016 |
| *Pinus taeda* | GCA_000404065 | KY964286 | NC_039746 | Zimin *et al.* 2017,  Asaf *et al.* 2018 |

# **Table S6.** Assembly and annotations statistics for all spruce and pine nuclear genomes used in phylogenetic and comparative genomics analysis

. NG50 was calculated using abyss-fac with -G 20e9. The fourth and seventh columns indicate the quality of the assemblies and annotations, respectively, as assessed by BUSCO. The second last column refers to the number of complete protein-coding sequences with lengths ≥ 1 kbp and intron lengths ≥ 10 bp.

| Species and genome assembly version | NG50 length (bp) | Reconstruction (Gbp) | Number of complete BUSCOs (-m genome) | Number of protein-coding sequences | Number of transcripts | Number of complete BUSCOs (-m protein) | Number of protein-coding sequences used for analysis | Reference |
| --- | --- | --- | --- | --- | --- | --- | --- | --- |
| *Picea mariana* v1 | 35,958 | 18.27 | 444 (27.4%) | 66,332 | 68,738 | 416 (25.7%) | 35,719 | This study |
| Interior spruce v5 | 121,714 | 20.14 | 796 (49.4%) | 28,943 | 58,801 | 291 (18.0%) | 28,878 | Gagalova *et al.* 2022 |
| *Picea abies* v1 | 500 | 9.93 | 570 (35.3%) | 70,736 | 178,049 | 176 (10.9%) | 23,233 | Nystedt *et al.* 2013 |
| *Picea engelmannii* v1 | 355,449 | 20.75 | 736 (45.6%) | 35,226 | 60,224 | 283 (17.6%) | 35,226 | Gagalova *et al.* 2022 |
| *Picea glauca* v2 | 131,339 | 21.58 | 784 (48.6%) | 30,140 | 55,309 | 290 (18.0%) | 30,140 | Gagalova *et al.* 2022 |
| *Picea sitchensis* v1 | 38,458 | 18.22 | 603 (37.4%) | 30,324 | 57,709 | 294 (18.2%) | 30,324 | Gagalova *et al.* 2022 |
| *Pinus lambertiana v1.5* | 2,702,679 | 19.82 | 1012 (62.7%) | 38,518 | 38,518 | 1,183 (73.3%) | 31,547 | Gonzalez-Ibeas *et al*. 2016 |
| *Pinus taeda v2.01* | 103,592 | 20.43 | 819 (50.7%) | 51,751 | 51,751 | 673 (41.7%) | 37,499 | Zimin *et al.* 2017 |

# **Table S7.** Summary of OrthoFinder results

. The longest transcript of complete protein-coding sequences with lengths ≥ 1 kbp and intron lengths ≥ 10 bp were passed as input to OrthoFinder.

| Species and genome assembly versions | Number of protein-coding sequences | Number of protein-coding sequences assigned to orthogroups | Number of orthogroups | Number of species-specific orthogroups | Reference |
| --- | --- | --- | --- | --- | --- |
| *Picea mariana* v1 | 35,719 | 32,936 | 19,330 | 560 | This study |
| Interior spruce v5 | 28,878 | 16,638 | 27,309 | 188 | Gagalova *et al.* 2022 |
| *Picea abies* v1 | 23,233 | 21,494 | 16,975 | 139 | Nystedt *et al.* 2013 |
| *Picea engelmannii* v1 | 35,226 | 33,104 | 18,180 | 253 | Gagalova *et al.* 2022 |
| *Picea glauca* v2 | 30,140 | 28,696 | 16,852 | 269 | Gagalova *et al.* 2022 |
| *Picea sitchensis* v1 | 30,324 | 28,381 | 17,661 | 197 | Gagalova *et al.* 2022 |
| *Pinus lambertiana v1.5* | 31,547 | 29,395 | 18,006 | 793 | Gonzalez-Ibeas et al. 2016 |
| *Pinus taeda v2.01* | 37,499 | 33,764 | 17,772 | 1,164 | Zimin *et al.* 2017 |

**Figure S1.** Best matching PFAM domains (n > 5) in *Picea mariana* specific protein-coding sequences

with the number of different EggNOG functional categories annotated for each bar. Functional category annotations were assigned based on hits to entries in the EggNOG database, while PFAM annotations were assigned via hits in EggNOG and/or InterPro databases as part of the EnTAP annotation pipeline. Bars in grey represent sequences that were not assigned functional categories as they were annotated with InterPro hits only.
